# Single cell profiling of CD45^+^ spinal cord cells reveals microglial and B cell heterogeneity and crosstalk following spinal cord injury

**DOI:** 10.1186/s12974-022-02627-3

**Published:** 2022-11-04

**Authors:** Elizabeth S. Fisher, Matthew A. Amarante, Natasha Lowry, Steven Lotz, Farhad Farjood, Sally Temple, Caitlin E. Hill, Thomas R. Kiehl

**Affiliations:** Neural Stem Cell Institute, Rensselaer, NY 12214 USA

**Keywords:** Spinal cord injury, Microglia, Single cell RNA-sequencing, Ectopic lymphoid follicle, B cells, Cell–cell communication

## Abstract

**Background:**

Immune cells play crucial roles after spinal cord injury (SCI). However, incomplete knowledge of immune contributions to injury and repair hinders development of SCI therapies. We leveraged single-cell observations to describe key populations of immune cells present in the spinal cord and changes in their transcriptional profiles from uninjured to subacute and chronic stages of SCI.

**Methods:**

Deep-read single-cell sequencing was performed on CD45^+^ cells from spinal cords of uninjured and injured Swiss-webster mice. After T9 thoracic contusion, cells were collected 3-, 7-, and 60-day post-injury (dpi). Subpopulations of CD45^+^ immune cells were identified informatically, and their transcriptional responses characterized with time. We compared gene expression in spinal cord microglia and B cell subpopulations with those in published models of disease and injury. Microglia were compared with Disease Associated Microglia (DAM) and Injury Responsive Microglia (IRM). B cells were compared to developmental lineage states and to an Amyotrophic Lateral Sclerosis (ALS) model.

**Results:**

In uninjured and 7 dpi spinal cord, most CD45^+^ cells isolated were microglia while chronically B cells predominated. B cells accumulating in the spinal cord following injury included immature B to mature stages and were predominantly found in the injury zone. We defined diverse subtypes of microglia and B cells with altered gene expression with time after SCI. Spinal cord microglia gene expression indicates differences from brain microglia at rest and in inflammatory states. Expression analysis of signaling ligand–receptor partners identified microglia–B cell interactions at acute and chronic stages that may be involved in B cell recruitment, retention, and formation of ectopic lymphoid follicles.

**Conclusions:**

Immune cell responses to SCI have region-specific aspects and evolve with time. Developmentally diverse populations of B cells accumulate in the spinal cord following injury. Microglia at subacute stages express B cell recruitment factors, while chronically, they express factors predicted to reduce B cell inflammatory state. In the injured spinal cord, B cells create ectopic lymphoid structures, and express secreted factors potentially acting on microglia. Our study predicts previously unidentified crosstalk between microglia and B cells post-injury at acute and chronic stages, revealing new potential targets of inflammatory responses for SCI repair warranting future functional analyses.

**Supplementary Information:**

The online version contains supplementary material available at 10.1186/s12974-022-02627-3.

## Background

Spinal cord injury (SCI) initiates complex molecular cascades signaling both tissue destruction and remodeling [1, 2]. The cellular and molecular mechanisms leading to persistent deficits after SCI are only partially understood. Recently, profiling of individual cells within the spinal cord and their temporal responses after SCI by single-cell RNA sequencing (scRNA-seq) has substantially advanced our understanding of the breadth of cellular plasticity following SCI, and revealed concurrent dynamics within specific cell types including neurons, glia, and immune cells [3–7]. A recent scRNA-seq study of immune cells in severe SCI showed prolonged gene expression changes in microglia (up to 3 months post-injury), which were proposed to enhance recovery [4]. The interactions between different immune cell classes, especially in chronic injury, remain largely unknown.

The immune response is a key mediator of tissue damage and wound healing. It intricately coordinates cellular responses of both the innate (e.g., neutrophils, macrophages) and adaptive (e.g., T cells, B cells) immune systems. After SCI, microglia, the central nervous system (CNS) resident immune cells, and infiltrating immune cells become activated and initiate diverse and complicated damage responses [8]. Prior studies have examined individual immune cell classes and their responses following SCI [9–14] including neutrophils [15–17], macrophages [18, 19], T cells [20, 21], and B cells [22]. SCI fails to elicit coordinated immune cell responses necessary for effective tissue repair [23] leading to chronic inflammation, poor wound resolution, and tissue fibrosis [2, 19, 24–26]. To effectively develop therapeutic interventions to mitigate SCI tissue damage, prevent chronic inflammation, and initiate effective remodeling, a more complete temporal understanding of the immune response and interactions occurring between infiltrating and resident immune cells is required.

In the current study, we profiled resident and infiltrating CD45^+^ cells in the intact and injured spinal cord at subacute and chronic stages using scRNA-seq to establish how the various immune cells change and respond to the evolving injury. The Takara ICell8 3' DE single cell system (chip/well-based), based on the SMART-seq cDNA synthesis chemistries, has advantages in library efficiency and a higher fraction of cell-related reads [27, 28]. This method yields increased sensitivity to rare transcripts compared to droplet-based approaches. It also allows for robust cell population identification with fewer cells than required for droplet based scRNA-seq [27–30]. CellPhoneDB was used to probe for potential cell–cell interactions warranting further exploration as targets for SCI repair [61]. Based on the gene expression profile of individual immune cells, we show that SCI elicits pronounced changes in microglia and B cell numbers and functions. Microglia undergo SCI-specific gene expression changes distinct from those occurring following brain injury, creating a computationally identified population appearing acutely and persisting chronically. SCI causes formation of ectopic lymphoid follicles at the injury epicenter containing immature and maturing B cells. The microglial and B cell responses arising following injury and persisting chronically include the expression of multiple chemokine receptor–ligand pairs indicating cellular crosstalk laying the groundwork for future empirical studies. Together, our results reveal that SCI induces chronic gene expression modifications in both resident and infiltrating immune cells. This resource identifies persistent cell–cell interactions between and within B cells and microglia that can be mined and tested to address their role in pathobiology and chronic degeneration seen in the spinal cord following injury.

## Materials and methods

### Animals

The care and use of animals were approved by University at Albany Institutional Animal Care and Use Committee and overseen by the Animal Resources Facility at the University at Albany East Campus Animal facility.

### Spinal cord injury

Adult (10–12-week-old) female Swiss Webster mice (Taconic) were housed in a pathogen-free barrier facility and maintained on a 12-h dark/light cycle with food and water provided ad libitum. A moderate (6.25 mm height) contusion SCI was performed using the NYU Impactor device. The mice were deeply anesthetized using isoflurane (3%). Following a 1.5–2 cm medial skin incision, the skin was loosened from the underlying tissue with a sterile cotton tipped applicator. Thoracic vertebrae (T9) was then identified, and the spinalis and semispinalis muscles on either side around T9 were cut with microscissors. The spinous processes and lamina of T9 and part of T8 and T10 were then exposed by removing any remaining semispinalis muscle using fine forceps and a T9 laminectomy was performed. The laminectomy site was then visually inspected to ensure that there was no damage to the spinal cord prior to placing the mouse on a platform beneath the NYU impactor. The impactor probe (1 mm diameter) was lowered to just above the spinal cord and centered on the spinal cord midline before raising it to 6.25 mm and inducing the spinal cord contusion. Following injury, 3.0 chromic gut sutures were used to reapproximate the muscles and overlaying skin in two separate layers. Topical analgesic, 2% lidocaine gel (McKesson #495443), was applied to the incision site. Buprenex (0.2 mg/kg) (Patterson Veterinary #433502), Baytril (5 mg/kg) (Patterson Veterinary # 07-89106228), and Lactated Ringer's solution were administered subcutaneously for analgesia, antibiotic, and hydration control, respectively. The mice were maintained on heating pads to regulate their temperature until fully awake before being returned to their home cages. Additional Buprenex was administered twice daily for three days for postoperative pain control and antibiotics were given once a day for five days. Bladders of injured mice were expressed twice daily. At three, seven, and 60 days after SCI, animals were sacrificed by decapitation under deep anesthesia with 150 mg/kg intraperitoneal pentobarbital (Merck #4199).

### Flow cytometry

Following sacrifice, animals from all groups (*n = 24, n* = 6/timepoint) had a 3-mm section of spinal cord dissected out centered at T9. Meninges were removed from the spinal cord segments under a dissecting scope. The spinal cord segments from six mice per timepoint were pooled and minced in ice-cold Hank's Balanced Salt Solution (HBSS). Following enzymatic dissociation with trypsin (0.25 mg/mL in DMEM) at 37° for 20 min, the cells were triturated with a Pasteur pipette to further dissociate the cells and then passed through a 40 μm cell strainer to reduce myelin debris. Trypsin was inactivated by the addition of DMEM + 10% Fetal Bovine Serum (FBS). Isolated single cells were then centrifuged and washed twice with fluorescence activated cell sorting (FACS) buffer (phosphate-buffered saline [PBS], 2% bovine calf serum). This was followed by a 30 min incubation with antibodies against CD45 (FITC, BD Biosciences #553079) diluted 1:20 in FACS buffer, shielded from external light. The stained cells were then washed twice with PBS. Flow cytometric analysis was performed using a BD FacsAria Flow Cytometer/Cell Sorter. Sorting gates were determined based on cells from unstained, uninjured control animals. This strategy allowed us to focus on smaller, more compact cells that were more likely to include microglia (see Additional File 1). CD45^+^ hi and lo cell populations were identified, and both were collected into a 1.5 ml tube containing DMEM for subsequent scRNA-seq analysis. Gating selection for the CD45^+^ immune cells of the spinal cord is illustrated in Additional file 2. Doublet discrimination was performed twice to maximize the isolation of single cells before dispensation onto the chip.

### ICell8 preparation and dispense

Cells were prepared according to the ICell8 manufacturer's protocol prior to single-cell dispense. Briefly, the total cell number obtained from FACS was used to calculate a dilution to dispense 1400 cells per well into a 384-well plate. Cells were stained with NucBlue (identifies cell nuclei during the imaging step) at a concentration of 2:25 and Propidium Iodide (to identify dead cells) at a concentration of 1:25 for 20 min at 37 °C (ThermoFisher Cell Viability Imaging Kit #R37610). Cells were centrifuged for 5 min at 300 *g*, and supernatant was discarded. Cells were resuspended in 80 µL/well PBS. 1 µL/well of RNase (New England Biolabs # M0314L) was added, plus 1 µL/well ICell8 100X diluent. Eighty µL of resuspended cells was added to each of the designated sample wells of the ICell8 384-well plate. The 384-well plate and an ICell8 chip were loaded into the ICell8 machine, and the standard dispense program was run to dispense 50 nL of sample into each well of the chip (total 5184 wells), in addition to fiducials (dye used to center the well for the imaging step for cell selection) and RNA controls (Takara #636643).

### Chip imaging and cell selection

**Fig. 1:**
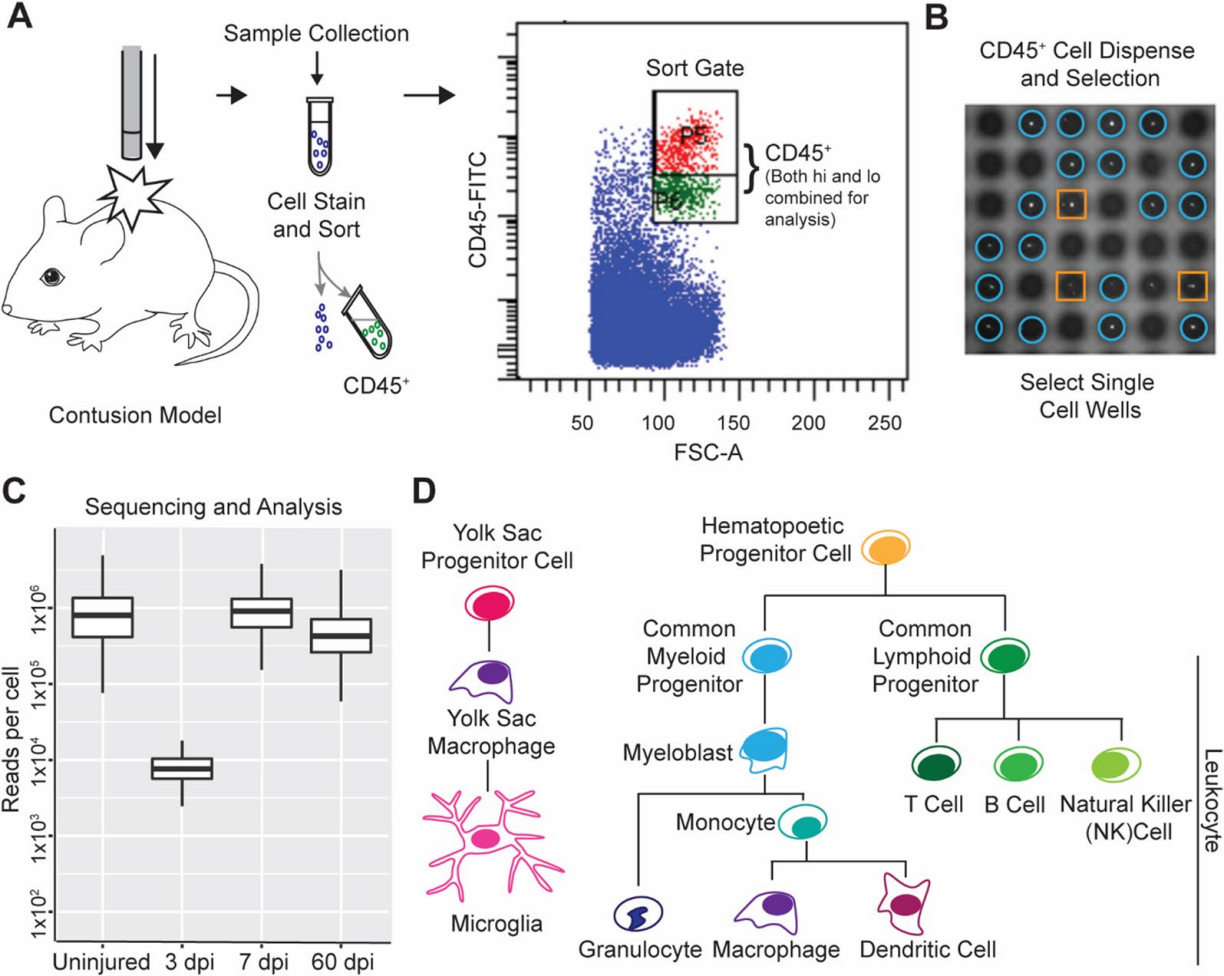
Workflow and quality control for single-cell isolation and sequencing of spinal cord immune cells. **A** Schematic of experimental approach. Spinal cords were harvested from mice, dissociated, and then stained for CD45 before being run on a FACSAria for sorting. Red and green cells identifying ‘hi’ and ‘lo’ expressing CD45^+^ cells, were sorted together for subsequent analysis. **B** Isolation of single cells onto the ICell8 chip. Wells containing single live cells (blue circles) were identified by microscopy imaging for further processing; wells with multiple cells or dead cells (orange squares) were excluded. **C** Reads per cell for the four conditions, represented by a box and whiskers plot. Box represents median and quartiles, while the whiskers are maximum and minimum values, excluding outliers. **D** Schematic of the immune cell lineage, simplified to include cell populations relevant to this study

After dispensing, the chip was blotted with filter paper and sealed with imaging film. The chip was centrifuged at 300 *g* for 5 min and set on the microscope. Fiducials were confirmed and images acquired via ICell8 Image software. Once images were acquired, the chip was placed in a chip holder and frozen at −80 °C. Single, live cells were selected for further processing based on imaging for NucBlue/propidium iodide stain using ICell8 CellSelect software (Fig. 1C).

### Single-cell RNA library preparation and sequencing

From uninjured control, 3 dpi and 7 dpi, 500 spinal cord immune cells were selected from each chip as single cells. Five K562 cells (control cells provided with the system), five negative control wells (no RNA, but all ICell8 buffers), and five positive control wells (12 pg RNA) were also identified. For 60 dpi, 1000 spinal cord immune cells were isolated and dispensed in a similar manner.

Libraries were prepared according to the ICell8 single-cell SMART-seq 3’ DE library preparation kits (Takara #635040) and were sequenced (150 cycles) on the NextSeq500 platform (Illumina) lane (4 × 10^6^ potential total reads).

A Bioanalyzer was used to assess the quality of the resulting cDNA and to monitor the size distribution of the transcripts. From our samples we obtained approximately 7–8 ng of cDNA with an average of 1650 base pair fragments, indicating good quality sampling of the immune cell RNA from the uninjured and injured spinal cords, enabling us to proceed to sequencing of the cDNA.

### Analysis of single-cell RNA sequencing data

Data were initially processed using Bcl2fastq (Illumina) to convert raw BCL files to fastq. Fastq files were split based on single-cell barcodes and ICell8 well list files using in-house java and R code, which is available on github [31]. Reads were mapped to mouse genome version mm10 using STAR aligner (v2.5) [32]. Aligned reads were sorted and aggregated using SAMtools and raw counts determined using Bioconductor packages in R [33, 34].

Following the well documented workflows of the Seurat3 package [35] we normalized, scaled, transformed, integrated, and performed cell-cycle correction on the raw read data. Subsequently, the Seurat3 library was used to perform dimensional reduction, neighbor-finding, clustering and then to identify marker genes for the resulting clusters. Markers were computed for each cluster relative to the complete data set via the FindAllMarkers method. Pairwise markers were computed, using Seurat’s FindMarkers method, to compare clusters determined to be comprised of subtypes of the same general cell identity. The SingleR and hypeR annotation and enrichment tools were used to determine functional enrichment of marker genes and top-expressing genes by cluster and by timepoint [36, 37].

#### Gene ontology enrichment analysis

Differentially expressed genes were identified computationally in the Seurat package. Top expressing genes from timepoints and from specific cell-type clusters were then used to determine enriched gene ontology (GO) categories. These gene lists were processed using Gprofiler2 and REVIGO [38, 39] to calculate the GO enrichments. Enrichments and plots generated to show changes in GO terms over time were performed using the Screp function[40].

#### Integrated analysis of microglia with data from Hammond, et al. [41]

Whole brain control and lysolecithin stimulated data sets published by Hammond et al. [41] were retrieved from the Gene Expression Omnibus (GEO) (accession: GSE121654). The Seurat library was used to integrate these data, along with the data from our study, using the SCTransform library (which performs normalization, variance stabilization, and feature selection based on a UMI-based gene expression matrix). After this normalization, SCT dimensional reduction and clustering and cluster marker identification was performed using the well documented methods RunUMAP, FindNeighbors, FindClusters and FindMarkers from the Seurat library.

#### Integrated analysis of B cell subtypes with published lineage data [57]

Cells initially identified as B cells were further analyzed independent  from the remainder of our data set to identify their developmental linage. The SingleR Bioconductor package [36] was used to create a reference data set from GEO (accession: GSE74290). The SingleR methods were then applied, following documented workflows, to label B cells from our study relative to the reference data set we constructed. To highlight the most stable labels, a range of score cutoffs were evaluated and a score cutoff of 0.125 was selected to indicate the most stable B cell subtype labels generated in the reference.

#### Integrated analysis of B cell subtypes with meningeal B cell data [42]

Data published in GEO by Cohen et al. [42] (accession: GSE160193) provided another reference data set for the determination of potential labels for the B cells collected in this study. Sample GSM4862209 was excluded as its smaller scale severely limited the number of integration anchors that could be calculated during this integration. Cells were integrated into a single combined Seurat object along with all the cells collected in our study. The Seurat library was used to integrate these data following a process of SCT normalization followed by dimensional reduction and clustering. The relative location of labeled cells from Cohen [42] (“B” and “pre B”) and the B cells from this study (Additional file 3) were observed via Uniform Manifold Approximation and Projection (UMAP) to infer likely cell-type convergence.

### Integrated analysis of B cell subtypes with TabulaMuris data

Immune-relevant data subsets were collected from the *TabulaMuris* Consortium data [43]. This included both droplet and FACS sorted cells from spleen, marrow, brain, fat, limb, liver, and lung. The data were normalized and combined into one Seurat object using a process of finding the top 2000 variable features and 20 integration anchors. Smaller data sets from *TabulaMuris* cohort constrained the total number of reference anchors that could be utilized in this calculation. Seurat methods FindTransferAnchors and TransferData were used to find anchors and transfer labels to predict labels for our B cells.

### Spinal cord staining

Spinal cord tissue sections were kindly provided by Drs. John Gensel and Andrew Stewart from spinal cords harvested and sectioned from a previous study [44]. Briefly, male and female C57/BL6 were given a 60 kDyn SCI under ketamine (100.0 mg/kg) and xylazine (10.0 mg/kg) anesthesia using the Infinite Horizons Impactor (Precision Systems Instrumentation, LLC, Fairfax Station, VA). Mice were anesthetized using an overdose of ketamine and xylazine and perfused using cardiac puncture with 0.1 M PBS, then with 4% formaldehyde in PBS. Spinal cords were extracted, then post-fixed for 2 h at room temperature and washed in 0.2 M phosphate buffer overnight. Spinal cords were transferred to a 30% sucrose solution for 1 week before blocking in optimal cutting temperature compound (OCT; Thomas Scientific). Sections (10 μm thick) were cut in the coronal plane between   on a cryostat and stored at −20 °C.

Prior to staining, slides were dried at room temperature for 1 h. Tissue sections were then permeabilized for 1 h and blocked in PBS with 5% normal goat serum, 3% BSA, and 0.03% Triton-X 100. The background was quenched using TrueBlack (BioTium # 23007) according to manufacturer instructions. B cells were stained for CD45RA (BioRad # MCA1258GT) diluted 1:100 and neurons for Tuj1 (BioLegend #801202) 1:500 in the same blocking solution and incubated overnight at 4 °C, followed by three washes with PBS. Secondary antibodies (Jackson Labs #712–545-153 (B cells), Invitrogen #A21135 (neurons)), were added at 1:500 in blocking solution for 2 h at room temperature followed by three washes with PBS. DAPI (Invitrogen #D1306) was added 1:1000 in water for 5 min, followed by two washes in PBS. Tissue was mounted in Fluoromount (Sigma #F4680). Tissue was then imaged using a Zeiss LSM 780 or a Zeiss Observer D1.

## Results

### Single-cell sequencing of CD45^+^ cells after spinal cord injury

**Fig. 2:**
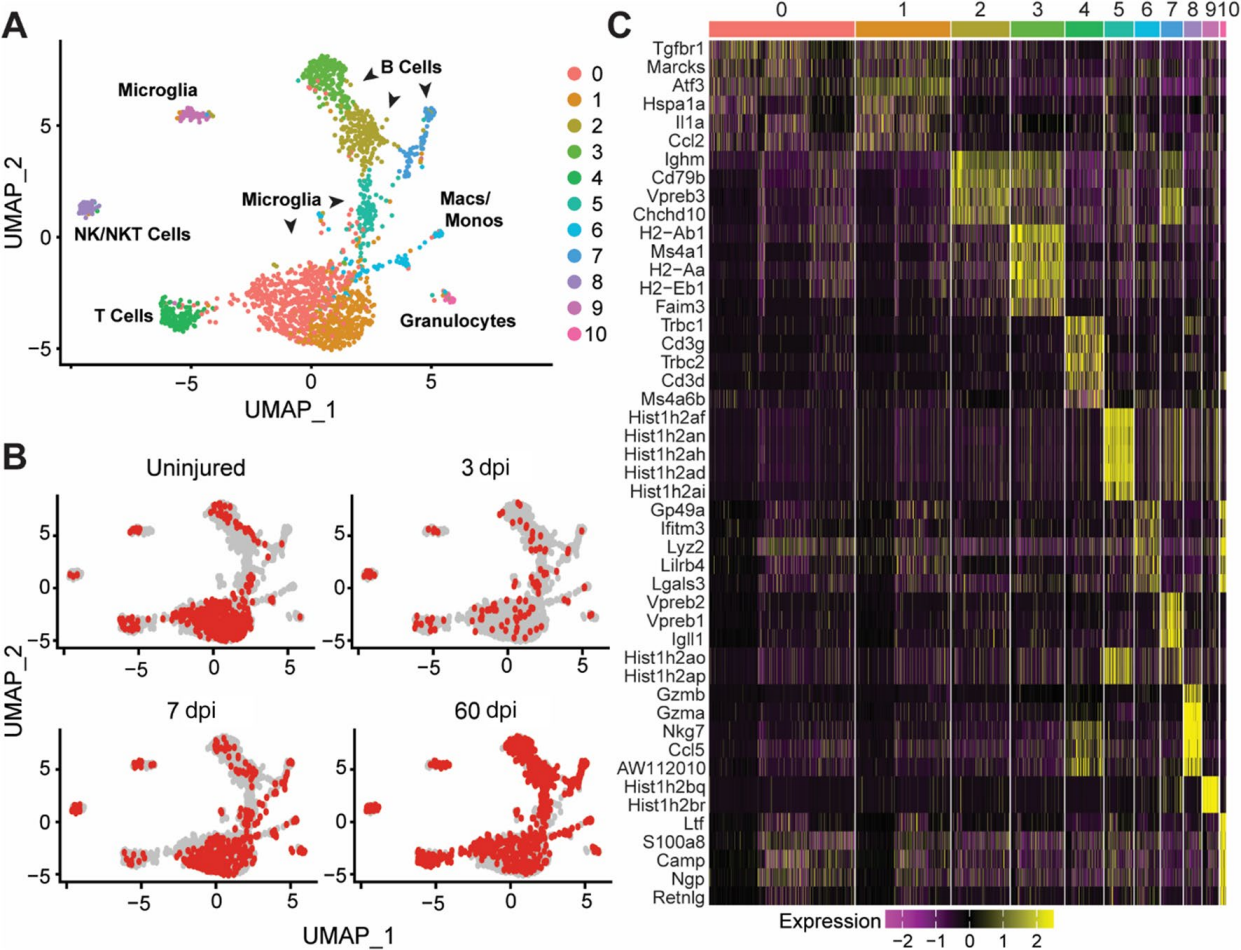
Analysis of combined data across all timepoints reveals clusters containing major immune cell types. **A** UMAP embedding of all timepoints combined after normalization by SCTransform. Clusters clearly separated into distinct immune cell populations. Clusters 0, 1, 5 and 9 express canonical microglia markers. Cluster 2, 3 and 7 express B cell markers. Cluster 4 expresses markers of T cells, and cluster 8 expresses NK and NKT (simplified hereafter as NK-/T) cell markers. Cluster 6 expresses markers of monocytes/macrophages, and cluster 10 expresses granulocyte markers. **B** UMAP plots highlighting the localization of cells across timepoints. Red cells are the cells isolated on the day indicated. **C** Heatmap of the top five genes driving cluster separation. *Macs* macrophages, *Monos*  monocytes, *NK* Natural Killer cells, *NKT* Natural Killer T cells

To establish the cellular and transcriptional profile of resident and infiltrating immune cells in the spinal cord, adult (10–12-week-old) female Swiss-Webster mice received a moderate thoracic spinal cord contusion injury (T9, 6.25 mm, NYU device). Moderate contusion injuries allow for the recruitment of immune cells following SCI and for some level of motor recovery in the animals [45]. Three-millimeter blocks of spinal cord tissue centered on the lesion epicenter were pooled, dissociated, and processed from injured versus uninjured control mice (*n* = 6 mice/condition), as summarized in Fig. 1A–C. Individual immune cells were isolated by FACS using the pan-immune cell marker CD45, focusing specifically on cells that were smaller and less granular by flow cytometry (i.e., reduced side scatter, Additional file 1). scRNA-seq was performed using the nanowell-based ICell8 system (Takara Bio USA), which generates more reads per cell than a droplet-based system. In total, 426 cells from uninjured, 102 cells from 3 dpi, 454 cells from 7 dpi, and 945 cells from 60 dpi passed quality control and were analyzed. The average total transcript reads across conditions was 645,000 reads per cell (Fig. 1C). Sample reads at each timepoint were well within the range necessary for downstream batch correction and analyses to robustly discriminate different cell populations [46]. Fewer cells and substantially fewer reads (median of 5,423 reads/cell) were detected in the 3-dpi sample compared to the others. While this did not affect the analysis in context of the whole data set, disparate read counts and sparsity from this sample introduced excess variability into downstream analysis of smaller subsets. As such, after the initial clustering analysis (Fig. 2), the 3 dpi data were excluded from subsequent analyses.

### Characterization of immune cell diversity after SCI

Samples from all timepoints were combined and clustered together using the shared nearest neighbor algorithm to gain an overview of the complete immune cell data set. Eleven cell clusters (Clusters 0–10) were identified and subsequently mapped to specific immune cell types (UMAP: Fig. 2A). The identified cell clusters included the major innate and adaptive immune cell types known to participate in the response to SCI [24, 47] including microglia (clusters 0, 1, 5, and 9), NK-/T cells (cluster 8), macrophages/monocytes (cluster 6), granulocytes (cluster 10), T cells (cluster 4) and B cells (clusters 2, 3, and 7) (Fig. 2A). Following SCTransform [48] to normalize the data and remove technical artifacts, samples were plotted by time to demonstrate proper normalization (Fig. 2B). The top five DE genes for each cluster are presented as a heatmap (Fig. 2C). Cell-type designations were first established by analyzing differentially expressed (DE) genes in each cluster and manually comparing them to several canonical markers of leukocytes and microglia. Cell-type designations were confirmed using both SingleR [36], a nearest neighbor, reference-based, label-transfer approach based on Spearman correlations, and by comparing the results with the cell-type reference ImmGen database (Additional file 4A, B).

### Dynamic temporal changes in immune cell types occur after SCI

**Fig. 3:**
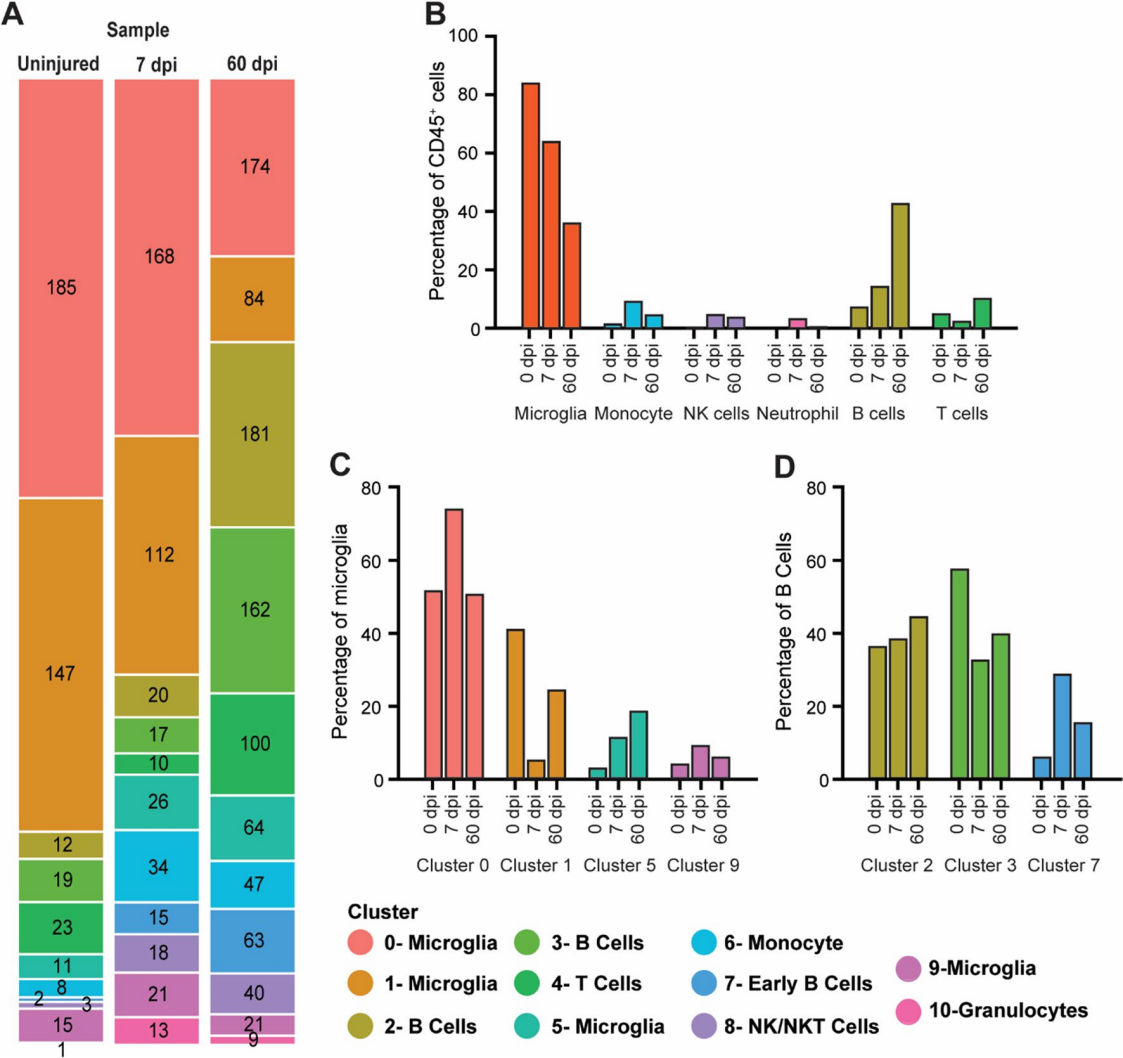
Immune cell populations isolated within the spinal cord change over time in response to SCI. **A** Stacked bar chart showing the number of cells present within each cluster at each timepoint as a proportion of all CD45^+^ cells isolated. **B** Bar chart of the percentage of major immune cell types present in the spinal cord and how they change in response to injury. **C**, **D** bar charts depicting the percentage of microglia (**C**) and B cells (**D**) in each cluster relative to all microglia or B cells, respectively. Note the change in percentage of the different clusters within each cell type in response to injury

Microglia (Cluster 0, 1, 5 and 9) and B cells ([2, 3 and 7]) comprised the bulk of the total CD45^+^ cells combined across timepoints (Fig. 3A, B) (53.8% and 28.5% of the total cells, respectively). The remaining 17.7% of total cells were macrophage/monocytes (5.2%), NK-/T cells (3.5%), neutrophils (1.3%) and T cells (7.7%). Our gating strategy focused on smaller, less granular, cells enabling us to capture microglia and to limit the number of granulocytes, macrophages and monocytes sorted for analysis.

The profile of the immune cells within the spinal cord changed in response to injury progression. In the uninjured spinal cord, the vast majority of CD45^+^ cells isolated were microglia (84%). The next largest cell population was B cells (7.7%), which may have included some peripheral blood cells, since animals were not perfused before tissue harvest. Peripheral myeloid, NK-/T, and T cells made up the remainder (8.3%). After SCI, microglia predominated sub-acutely (64.1% at 7 dpi); but, in chronic SCI, they comprised just 36.3% of total CD45^+^ cells. In contrast, B cells that were initially 7.7% of total CD45^+^ cells in the uninjured tissue increased to 14.7% at 7 dpi and became the largest population (43%) in the chronic state. Other cells identified showed smaller changes in response to injury (Fig. 3A, B). T cells decreased from ~ 5% of CD45^+^ cells in the uninjured cord to 2.8% at 7 dpi, rebounding to 10.6% of cells isolated chronically. Peripheral myeloid and NK-/T cells were present at all timepoints post-SCI, ranging from 2 to 10% for monocytes/macrophages, 0–4% for granulocytes, and 1–5% for NK-/T cells. Collectively, this analysis revealed substantial alterations in immune cell populations isolated within the acutely and chronically injured spinal cord, particularly in microglia and B cells.

Microglia and B cells were distributed across multiple clusters, implicating different cell activation states or subtypes[30]. Analysis of the proportion of microglia (Fig. 3C) and B cells (Fig. 3D) by their respective clusters showed time-specific changes warranting further analysis.

### Uninjured spinal cord microglia are primed to respond to injury

**Fig. 4:**
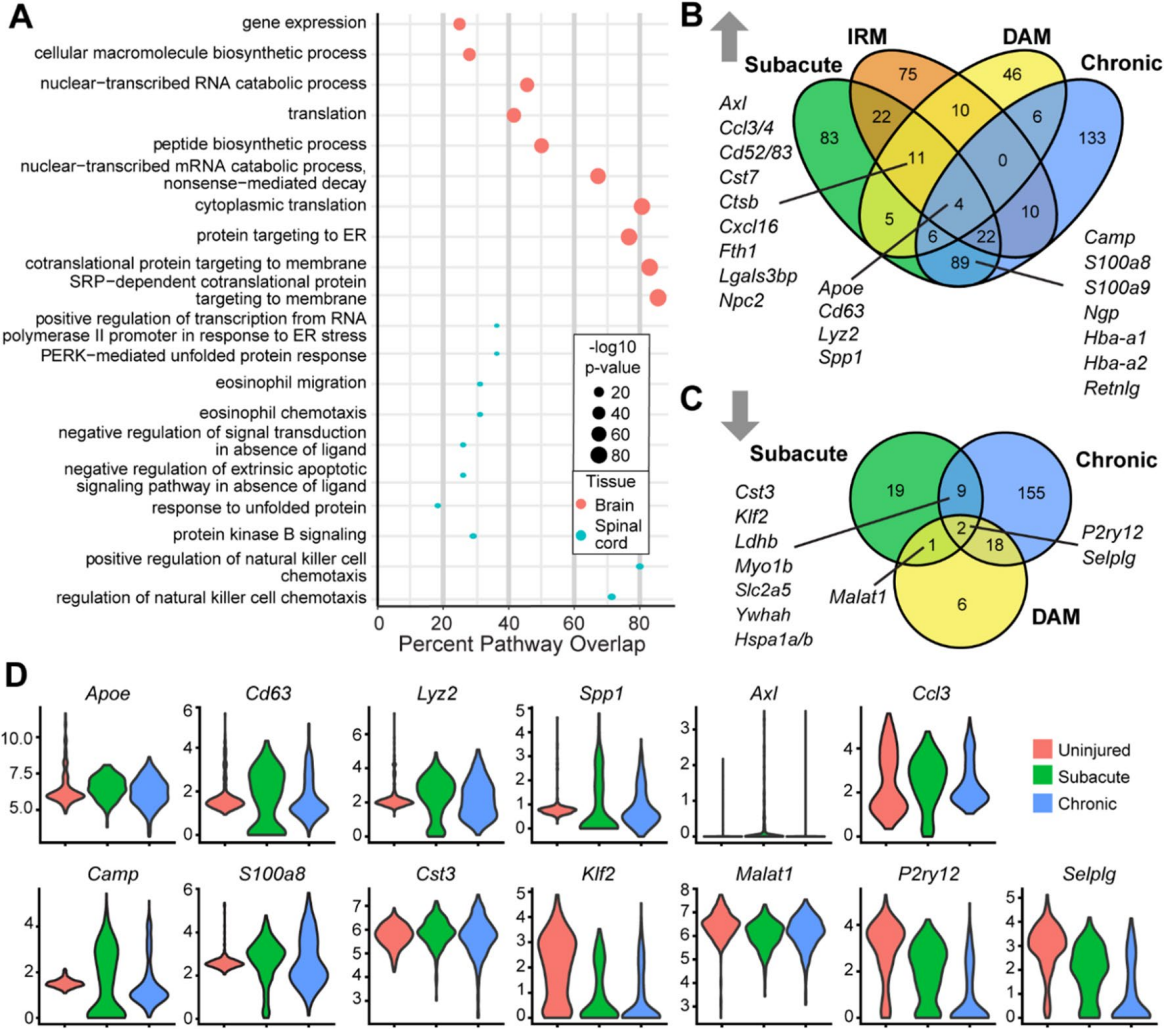
Comparison of uninjured and injured SCI microglia to uninjured, injured, and diseased brain microglia. **A** GO pathway enrichment in uninjured spinal cord and brain microglia [41] using significantly DE genes, represented as a dot plot (See Additional file 5 for additional enrichments). The x axis is the percentage of genes represented in the pathway which overlapped with enriched genes. **B** Venn diagram of genes significantly upregulated in the subacute and chronic phases of SCI versus Injury Response Microglia (IRM [41]) and Disease-Associated Microglia (DAM) [53] (See Additional file 6 for lists of up and down regulated genes following SCI.) **C** Venn diagram of genes significantly downregulated in subacute and chronic SCI versus DAM. **D** Violin plots of select significantly altered genes overlapping across injury states in our SCI data

SCI induces robust inflammation and greater scar formation than a similar magnitude brain injury [49, 50]. Microglia are hypothesized to be involved, as microglia–astrocyte interactions drive glial scar formation [51, 52]. To identify potential functional differences between brain and spinal cord microglia, we compared our uninjured spinal cord microglial transcriptomes with previously published brain microglia transcriptomes [41]. Once the data sets were integrated, we performed DE analysis in Seurat. The resulting gene lists were then evaluated via gene set enrichment analysis, against GO categories, to determine enriched pathways within and across data sets. This identified several distinct gene expression profiles and GO pathway enrichments for microglia in each tissue (Fig. 4A, Additional file 5). Brain microglia were enriched for functions related to RNA processing, including translation, protein synthesis and protein processing. In contrast, spinal cord microglia were enriched for processes involved in peripheral immune cell recruitment and oxidative stress-related processes, such as "PERK-mediated unfolded protein response" and nitric oxide biosynthetic and metabolic processes. This analysis suggests that the brain and spinal cord microglia differ at rest, though more empirical evidence should confirm these identifications.

### Spinal cord and brain microglia respond differently to injury

Similarities between spinal cord microglia and disease associated microglia (DAM) [4] have recently been identified. Activated brain and spinal cord microglia were compared using up- (Fig. 4B) and down- (Fig. 4C) regulated microglial genes in subacute and chronic SCI to those previously identified in brain injury and disease. For brain microglia, we used signature genes previously established for DAM from Alzheimer’s Disease (AD) model mouse brains [53], and injury responsive microglia (IRM [41]), generated by injection of lysolecithin into brain white matter. DAM were collected from 6-month-old 5XFAD mice, which are on a C57/BL6-SJL background, and IRM were isolated from 100-day-old C57/BL6-JL mice 7 days following lysolecithin injection. Both studies were performed using droplet-based approaches. For down-regulated genes, the comparison was restricted to SCI-microglia and DAM, because downregulated IRM genes were not available for analysis.

Tissue- and temporal-specific changes in gene expression were identified for spinal cord and brain microglia in response to injury (Fig. 4B, C). Six overlapping genes were identified across all conditions. SCI–microglia shared four upregulated genes with DAM and IRM, *Apoe, Cd63*, *Lyz2*, and *Spp1* (Fig. 4D), and two downregulated genes with DAM, the canonical microglia markers [41, 53, 54] *P2ry12* and *Selplg* (Fig. 4D). Temporal evaluation post-SCI identified eleven upregulated genes in acute SCI shared with DAMs and IRMs. These genes were related to metabolism (*Cst7*, *Ctsb*, *Npc2*, and *Fth1*a), and cell migration/adhesion (*Ccl3*, *Ccl4*, *Cxcl16*, *Cd63, Lgals3bp* and *Axl*). They also shared one downregulated genes with DAM, *Malat1*. A subset of the common genes is shown in Fig. 4D. The DAM and IRM microglia genes overlapping with acute SCI included *Axl*, a gene upregulated in brain microglia in almost all disease states [55]. Chronic SCI–microglia had no upregulated genes in common with DAM and IRM, but eighteen downregulated genes were shared with DAM, including canonical microglia genes *Cx3cr1*, *P2ry13*, and *Csfr1*. This indicated that while some SCI–microglia transcriptional responses resemble those in the injured brain [4], most were unique to SCI microglia isolated in our study. This could contribute to altered injury magnitudes between different CNS sites, which should be tested empirically.

### Chronic response of B cells following SCI

The composition of B cells present after SCI, their functional profile, and the mechanisms leading to their accumulation are not yet known. The prevalence of B cells in our data set—25% of the total CD45^+^ cells across timepoints and 43% of cells isolated at 60 dpi—indicated roles for B cells in spinal cord pathophysiology beyond those currently described [22, 56].

**Fig. 5:**
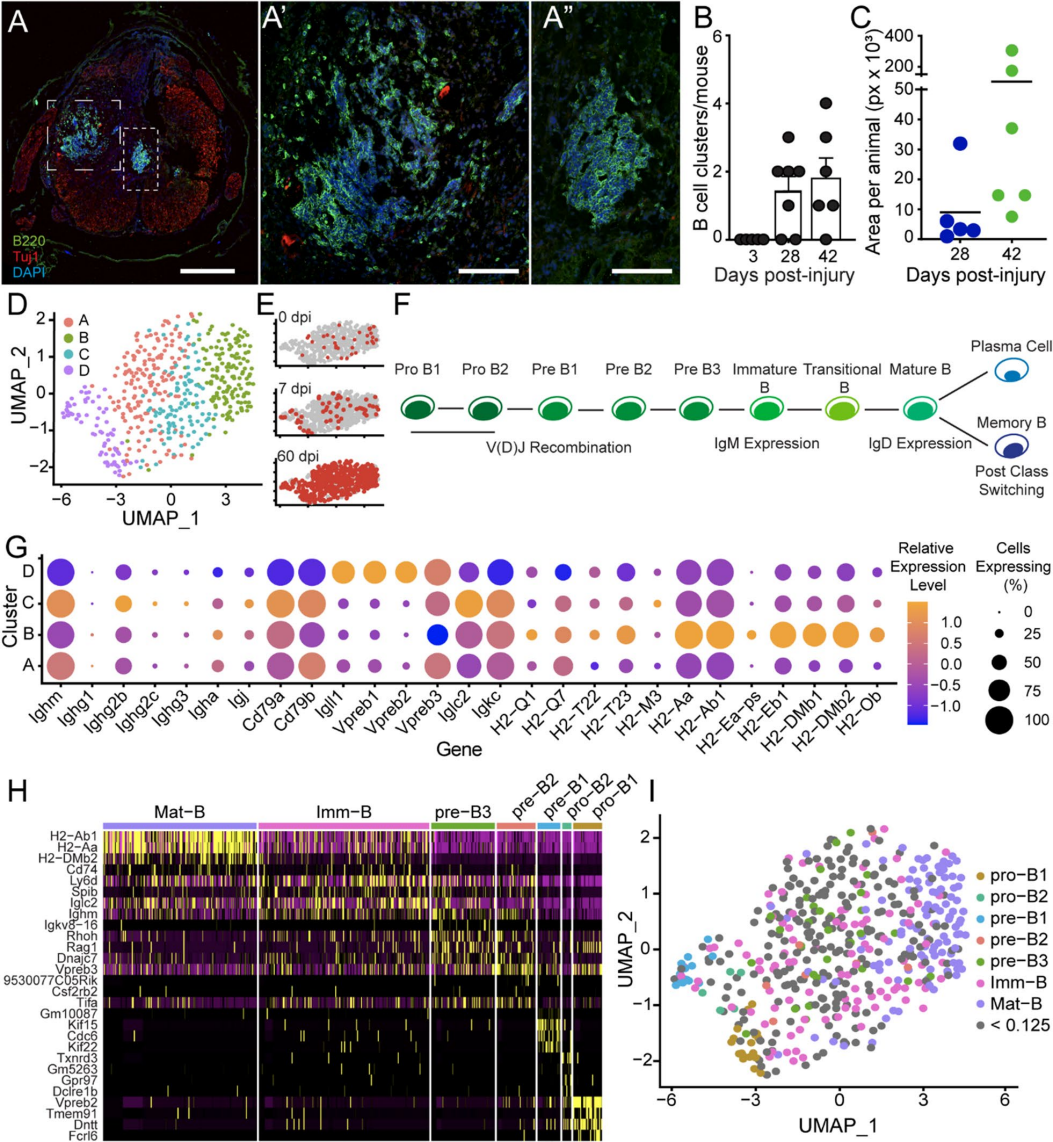
B cells increase in prevalence over time following SCI and contain multiple subtypes. **A** Immunohistochemical staining of B cells in spinal cord tissue 42 dpi, stained with B220 (Green), Tuj1 (Red), and DAPI (Blue). Large and small dashed boxes indicate the regions shown in high power in **A**’ and **A**,” respectively. Scale bars: **A** 500 μm, **A’** and **A”** 100 μm. **B** Quantification of number of clusters identified in SCI animals over time following injury. Each dot represents an individual animal. Error bars represent SD. **C** Quantification of the area of clusters per section, represented in pixels. **D** Isolated and re-clustered UMAP of clusters 2, 3, and 7 identifies four subclusters of B cells. **E** UMAP plot highlighting cells isolated from each timepoint, top is 0 dpi, middle is 7 dpi, and bottom is 60 dpi, demonstrating most B cells were isolated at 60 dpi. **F** Dot plot demonstrating relative abundance and expression of the Ig class detected, of selected markers of early B cells, and selected MHC-I and MHC-II genes. **G** Schematic showing the developmental lineage of B cells including important events along their development. **H** Heatmap of re-clustered data, and genes identifying them as a subtype identified by Jensen [57]. **I** UMAP of B cell clusters, with cells identified by developmental stage

First, we examined mouse spinal cord sections by immunohistochemistry for B220, a general marker of B cells. These sections came from the B57/BL6 strain, treated with a moderate thoracic contusion using the Infinite Horizon Impactor (for more details, see methods) [44]. B220^+^ cells were found clustered in large discrete foci within the spinal cord at 42 dpi, (Fig. 5A-A’’). B cells were found in the spinal cord at 3 dpi; however, they were present as individual cells, and no clusters were evident. Quantification of the number and area of clusters showed they formed between 3 and 28 dpi (Fig. 5B) and increased in size between 28 and 42 dpi (Fig. 5C, Additional file 7A, B). Similar accumulation of B cells with time post-SCI has been reported and hypothesized to contain of a mixed cell population [22, 56].

To profile the B cells further, a secondary cluster analysis was performed using the three original clusters (Clusters 2, 3, 7). Four distinct sub-clusters, denoted A–D, were delineated (Fig. 5D; Additional file 8; heatmap of top DE genes in the clusters)—including one, Cluster D, which emerged post-SCI (cf., Fig. 5D, E). B cell development involves progression from Pro-B1 cells through Mature B, which are activated to either produce antibodies (Plasma cells) or act as antigen presenting cells (APCs) (Fig. 5F). To establish the activation and maturation states of cells present, several analyses were performed.

Expression of components of the B cell receptor (BCR) were examined to identify maturation state and whether any B cells were acting as APCs. Components analyzed included immunoglobulin heavy (Igh) and light chains (Igl, Igk), signaling components Ig-a (Cd79a) and Ig-b (Cd79b), Ig subclass type (i.e., IgM, IgD, IgG, IgA, IgE), and expression of MHC-I and MHC-II (Fig. 5G). Cluster D, which appeared after SCI, had the most immature BCR. It contained cells expressing Igl components and enzymes needed for heavy chain V(D)J recombination, typically found in pro/pre B cells. Cluster A cells expressed intermediate levels of genes suggesting development of BCR expression. Cluster C cells expressed high levels of IgM and IgG2b indicative of activated B cells post-class switching. Cluster B cells expressed MHC-II components found in APCs. These results indicate the presence of B cells in multiple states of development and activation.

To identify their developmental state more precisely, our B cell data were compared to bulk RNA sequencing data from defined B cell linage states[57] made into a reference in SingleR[36]. This analysis identified different B cell developmental stages from Pro-B1 cells through Mature B cells (Fig. 5H). UMAP plotting of B cell identities showed Cluster D contained pro-B1, pro-B2, and pre-B1 cells, Cluster A was a mixture of pre-B2, pre-B3 and immature B cells, Cluster C included both immature and mature B cells and Cluster B was comprised of mature B cells (Fig. 5I). Thus, a range of the B cell lineage existed within the spinal cord (Fig. 5H, I), including a population of pro/pre-B cells that emerges after SCI.

Localization of pre-B cells to the spinal cord meninges was recently reported [42]. Comparison of our B cells with those isolated from the meninges of SCI and SOD1 mice [42], a model of amyotrophic lateral sclerosis (ALS), showed the B cells we isolated from the spinal cord were most similar to those found in the meninges under inflammatory conditions (Additional file 3A,B). This demonstrated immature B cells are not restricted to the meninges after SCI. Understanding the functions of the B cells within the spinal cord and factors driving their entry could lead to new interventions to counteract B cell-mediated spinal cord pathology [22, 56].

### Immune cell pathway responses after spinal cord injury

**Fig. 6:**
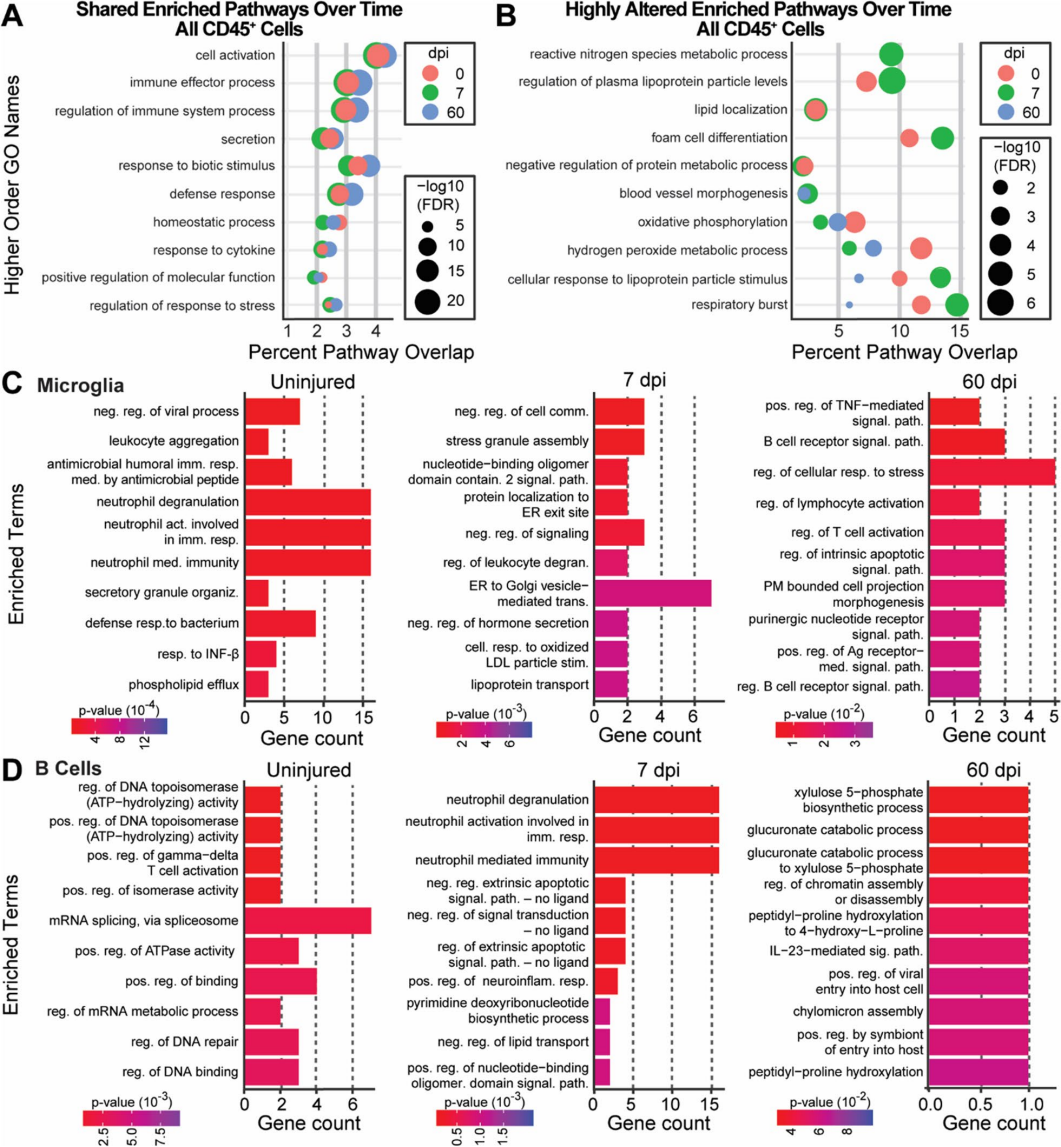
Identification of major pathways associated with timepoint and cell identity. **A**, **B** Per timepoint, the Wilcoxon rank sum test was used on all cells isolated, followed by GO enrichment using the hypeR package. Semantic similarities of enriched GO terms were generated using rrvgo package [90], producing new categories displayed as a dot plot. The size of the dot indicates the False Discovery Rate (FDR) for the enriched category, and the x axis indicates the percentage of genes overlapping that pathway (See also Additional file 9 for enrichments). **A** Enriched pathways with minimal change between timepoints. **B** Enriched pathways either changed with time or were not represented at all timepoints. **C**, **D** Wilcoxon rank sum test was used for microglia (**C**), or B cells (**D)** at each timepoint, followed by GO enrichment using the EnrichR package and the “GO Biological Pathway 2021 library” to generate bar plots of significantly enriched pathways at each timepoint for the individual cell types (See Additional file 11 for additional enrichments not included in bar charts).

SCI induces complex immune-mediated changes [58]. Using our scRNA-seq data set, functional analysis of the GO biological pathways and semantic similarity measurements were used to identify shared or divergent pathways across time in response to injury (Top 10 terms, Fig. 6A, B; complete data set, Additional file 9). We focused our analysis on the cell-type specific functional enrichments occurring in response to SCI for microglia and B cells. The functions of other immune cells are included in Additional file 10. Top enrichments unique for each cell type and timepoint analyzed indicates specific, evolving roles of the various immune cells after SCI.

Microglia are among the first cells to respond to injury, and have complex interactions with axons, glia and immune cells [25]. Our assessment showed that microglia in the uninjured, subacute–SCI, and chronic–SCI spinal cord had distinctly different gene expression across timepoints. The gene expression changes were suggestive of altered interactions between microglia and other immune cells (Fig. 6C). Uninjured microglia were enriched for terms reflective of a role in immune surveillance and interactions with neutrophils and other leukocytes; terms identified included “negative regulation of viral process,” “leukocyte aggregation,” and several associated with neutrophil biology. In subacute–SCI, microglia were enriched for pathways related to peripheral immune cell infiltration and stress responses including “regulation of leukocyte degranulation,” “negative regulation of cell communication,” “stress granule assembly,” and “lipoprotein transport.” In chronic–SCI, pathways linked to microglia–lymphocyte interactions predominated including “regulation of lymphocyte activation,” “B cell receptor signaling pathway,” “regulation of B cell receptor signaling pathway,” and “regulation of T cell activation.” Identification of terms in microglia related to their interactions with neutrophils and leukocytes aligns with their role as immune mediators between the CNS and periphery; currently, little is known about these interactions in the context of SCI. Detecting chronic microglia–lymphocyte interactions was particularly notable as it suggested a role for microglia in chronic B cell accumulation. Future work should further examine these results to confirm their involvement.

Unlike microglia, limited functions have been ascribed to B cells in the context of SCI [22, 56]. Our transcriptional analysis of B cells (Fig. 6D) indicated diverse roles related to antibody refinement and interactions with other immune cells. Uninjured spinal cord B cells were enriched for functions related to DNA and RNA modifications suggestive of antibody refinement, such as “regulation of DNA binding,” “regulation of DNA repair,” “regulation of DNA topoisomerase activity,” “positive regulation of isomerase activity,” and “mRNA splicing, via spliceosome.” Subacute SCI-B cells were enriched for multiple pathways regulating neutrophils, including “neutrophil degranulation,” “neutrophil activation involved in immune response,” and “neutrophil mediated immunity,” while chronic SCI-B cells were enriched for “interleukin-23 mediated signaling pathway” and several metabolism pathways. IL-23 signaling coordinates germinal center class-switching and promotes germinal B cell centers [59, 60]. Interestingly, both neutrophils and astrocytic-expression of IL-23 are linked to B cell accumulation and pathology in the experimental autoimmune encephalomyelitis (EAE) model of multiple sclerosis [60].

### Interactions between microglia and B cells in the spinal cord

To identify potential cell–cell interactions between immune cells, we applied the publicly available repository of curated receptors, ligands and their interactions, CellPhoneDB [61], to our data set. As we were interested in identifying potential mechanisms contributing to the accumulation of B cells within the chronic SCI to test in future studies, and microglia were by far the majority of cells in our data set, we focused on investigating microglia-B cells interactions; additional interactions are shown in Additional file 12.

**Fig. 7:**
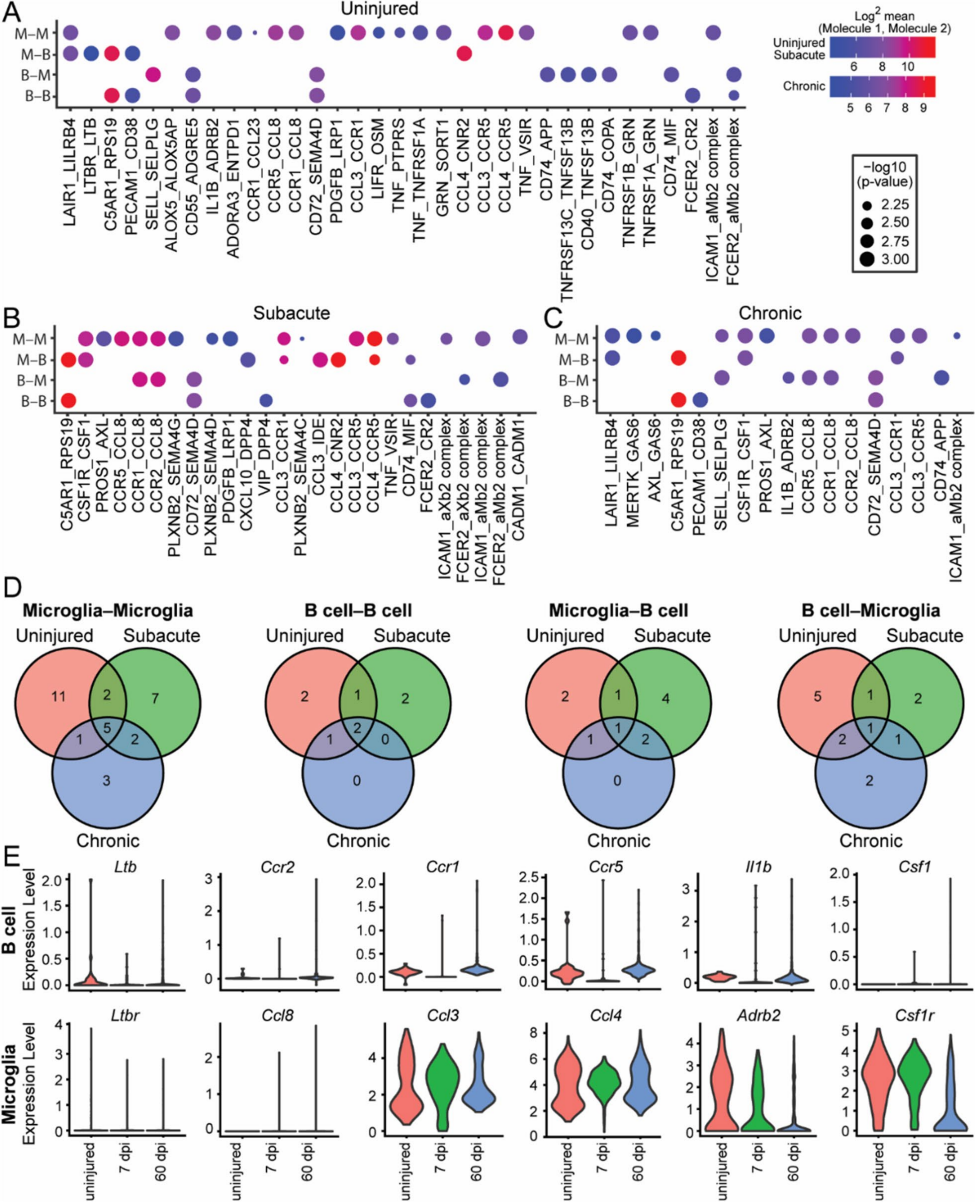
Cell–cell interactions between microglia and B cells in the injured spinal cord. **A**–**C** Microglia (clusters 0, 1 ,5 ,9) and B cell (clusters 2, 3 and 7) gene expression was analyzed at each timepoint [(**A**) uninjured, (**B**) subacute, (**C**) chronic] using CellPhoneDB [61] to determine genes involved in cell–cell interactions, shown as a dot plot. **D** Summary of the number of interactions within and across cell types and how they change over time using Venn Diagrams (See Additional file 14 for lists of these pairings). **E** Violin plots of selected genes discussed in text, and their expression in B cells or microglia

In total, 25 putative ligand–receptor interactions were identified between B cells and microglia (Fig. 7A–D). CellPhoneDB pairings are directional: 11 microglia-B cell (M–B) and 14 B cell–microglia (B–M) interactions were found. The number of predicted M–B/B–M pairings decreased with injury progression: 14, 12, and 9, were identified for uninjured, 7 dpi, and 60 dpi spinal cords, respectively. Examination of the identified receptor–ligand pairings predicts a subset with roles in microglial survival, immune cell infiltration, and neuroprotection warranting further investigation.

In line with functional identification of “negative regulation of viral process” in microglia from the uninjured spinal cord (Fig. 6C), one of the seven unique potential B-M/M-B interactions in uninjured spinal cord was the M–B interaction of lymphotoxin B receptor (*Ltbr*), and *Ltb* (Fig. 7A). In the lymph node, LTB–LTBR signaling between B cells and macrophages maintains macrophage responsivity to viral infections [62], and microglial LTBR has roles in de- and remyelination [63]. After SCI, 11 unique predicted interactions between microglia and B cells (M–B/B–M) were found (Fig. 7D) and notably, most were prevalent in subacute SCI (subacute, *n* = 6; chronic, *n* = 2; subacute and chronic, *n* = 3).

More than half of the interactions identified involved chemokines, potent attractants for peripheral immune cells to injury sites [23, 64]. Microglia expression of the chemokines C–C and C-X-C motif ligands (Ccl, Cxcl) *Ccl3*, *Ccl4*, *Ccl8* and *Cxcl10* comprised seven of the B–M/M–B interactions identified. In subacute SCI, interactions were found between microglia-expressing *Ccl8* and B cells expressing C–C motif chemokine receptors (Ccr) *Ccr1* or *Ccr2*, and between microglia-expressing *Ccl3* or *Ccl4* and B cells expressing *Ccr1* or *Ccr5* (Fig. 7B). In chronic SCI, the putative interactions for *Ccl3*- and *Ccl8*-expressing microglia and *Ccr1*- and *Ccr5*-expressing B cells persisted, and *Ccr1*- and *Ccr5* expression in B cells was augmented (Fig. 7E). *Ccl4* interactions were not observed in chronic SCI, but a new chemokine–receptor interaction emerged between *Ccl8*-expressing microglia and *Ccr5*-expressing B cells. The functional effects of these chemokines on microglia-B cell interactions after SCI remains to established. However, CCL8 is linked to leukocyte chemotaxis in other inflammatory conditions [65–67], and SCI-*Ccl3*-/- mice recruit fewer immune cells to the injury site [68], indicating microglia might attract B cells via CCL8 production; this and other B–M/M–B interactions will be worthwhile investigating in functional studies.

Interestingly, of B cell-related pathways in chronic microglia (Fig. 6C), only two of the identified potential interactions were unique to chronic SCI. One was *Ccr5*–*Ccl8*, discussed above, the other was an interaction between *Il1b* in B cells and *Adrb2* (adrenergic receptor β2) in microglia (Fig. 7C). In dendritic cells, *Adrb2* activation promotes production of anti-inflammatory lymphocytes [69], and in microglia it is neuroprotective [70–72]. One possibility is that chronic SCI microglia, rather than instructing B cells to invade or cluster as predicted to occur in subacute stages, are exchanging anti-inflammatory signals.

In line with possible protective roles for B cells in both subacute and chronic SCI, a possible M–B *Csf1r*–*Csf1* interaction was identified (Fig. 7B,C). Colony stimulating factor 1 receptor (CSFR1) signaling contributes to microglial survival [73]; and a study in humans found CSF1 is expressed by activated, but not resting B lymphocytes [74]. This suggests post-SCI, B cells produce CSF1 that promotes microglial health. Hence, the presence of activated B cells could be important for the long-term survival and maintenance of microglia after SCI.

Notably, by immunostaining we found B cells localized near lesion borders as identified by GFAP staining in chronic stages of SCI, at both 28 and 42 dpi (Additional file 13). This area has also been shown to harbor microglia at chronic injury stages [75], which may allow these cells to interact. We also observed individual B cells in the parenchyma in the chronic phase of SCI (Additional file 13), which were rare but could contribute to recovery or injury in the spinal cord. Future work should examine these interactions at these distinct locations in more detail.

## Discussion

Secondary injury propagated by immune cells leads to incomplete wound healing and chronic inflammation in the spinal cord. By performing scRNA-seq on CD45^+^ cells isolated from the adult mouse spinal cord in the uninjured, subacute, and chronic phases of SCI, we have revealed previously unappreciated diversity and changes in select immune cell subtypes over time post-SCI.

Peripheral immune cells increased significantly in the spinal cord following injury, including myeloid-derived-, NK-/T, T, and B cells. Except for myeloid cells, most peripheral cells peaked at 60 dpi, highlighting the importance of studying chronic inflammation in the spinal cord.

We restricted our examination to the subacute and chronic intervals post-injury and employed a gating strategy that excluded larger, more granular cells. Advantages of this approach include that it facilitated the identification of microglia, resulted in the identification of a large and diverse population of B cells, and allowed us to identify predicted interactions between microglia and B cells for subsequent experiments. One drawback of our method, and the restriction of our examination to 3 dpi and beyond, was that it resulted in isolation and identification of fewer cells of the myeloid lineage. Neutrophils and monocytes peak in the spinal cord within 24 h of SCI [3, 14, 76], and very few of these cells were captured in this study.

We focused on collecting microglia at the expense of neutrophils and macrophages. This ensured that we had sufficient cells to draw conclusions about the microglial responses with our chip-based approach. Several recent studies have focused on the immune cell response to SCI using scRNA-seq approaches using droplet-based approaches that allow for the analysis of a larger number of cells [3, 7, 42]. The study by Milich et al. [3], examined almost all cell types within the spinal cord at acute phases of injury, including 1, 3, and 7 dpi. Interestingly, most of our common upregulated microglia genes (*Apoe*, *Spp1*, and *Lyz2*, center of Venn diagram Fig. 4B) were also upregulated in Milich et al. [3], despite differences in the technologies used.

In addition to the enrichment of microglia, we isolated many B cells using our approach, which permitted us to examine their potential interactions after SCI. In the Milich study, they specifically excluded lymphocytes from their analysis. B cells have also been found in the other SCI studies in which the cellular changes have been explored by scRNA-seq [4, 7]. Our unique data set enabled us to investigate the potential interactions between these two cell types. Additional empirical studies are needed to assess the predicted interactions.

ScRNA-seq allows the identification of transcriptionally distinct populations of cells analyzed computationally. Populations identified rely on sampling criteria, sequencing modalities used, and downstream processing pipelines. Our chip-based sequencing method uses fewer cells but enables deeper sequencing (more reads-per-cell) than droplet-based methods. Our results may underestimate the full diversity of the immune cell response, as our samples were pooled to produce sufficient cells for analysis, thus masking animal to animal variations, and only a subset of immune cells were isolated by the gating strategy used. Nevertheless, we were able to isolate subpopulations that appear to have differential functions and distinct changes with time after injury.

At the single-cell level, microglia appeared abundant in the uninjured and sub-acutely injured spinal cord but were reduced in number chronically. While we collected several populations of microglia, we cannot exclude the possibility that we missed some low CD45-expressing microglia, as their expression of this marker is lower than macrophages [77]. However, our data integrate well with other scRNA-seq data sets of microglia [41, 42], which indicates we have good microglial representation.

We found in the uninjured state that microglia in the spinal cord demonstrated different pathway enrichments from those in the brain, and their gene expression response following SCI differed from the response of brain microglia to white matter injury or AD modeling. Such differences may help explain reports that similar magnitude injuries induce a less severe immune response in the brain compared to the spinal cord [50], though this would need to be further investigated. Our findings contrast with previous work showing gene expression similarities between SCI and DAM microglia [4]; this discrepancy may be due to many factors including injury severity, different sequencing platforms, the mouse strain used, or the cell selection strategy.

The largest change identified in response to SCI was the ~ sixfold increase in B cells isolated chronically compared to uninjured, which we found occurred predominantly within the injury site, as reported previously [22]. This was accompanied by an increase in B cell diversity: we observed multiple stages of the lineage including pro/pre B and mature B cells. Immature, actively rearranging pro/pre B progenitors were not observed in the uninjured state but appeared in the subacute phase following injury. The presence of proB2 cells could explain how deleterious antibodies against autoantigens are generated following SCI, as this is the stage when B cells are undergoing V(D)J recombination [56]. The expression of MHC-II and changes in Igh expression could participate in the formation of immune complexes linked to complement-mediated cytotoxicity within the chronically injured spinal cord [22]. At all stages, mature B cell genomic signatures were detected with diverse post-class switching immunoglobulins, as well as those expressing MHC-I and -II molecules.

Ectopic clusters of B cells form within the spinal cord in autoimmune disease [78], and can occur within the injured spinal cord parenchyma [22, 56] and meninges [42] and generate cytotoxic antibodies [22, 79]. It is tempting to suggest B cells are recruited during subacute SCI, and respond to injury by setting up ectopic lymphoid follicles that grow locally, producing B cells reacting to the injured environment by producing antibodies and other factors contributing to long-term inflammation [78]. Indeed, the shift from individual B cells present at 3 dpi, B cells coalescing in ‘ring-like’ structures at 28 dpi (Additional file 7C) to large foci at 42 dpi suggest these clusters grow from B cells initially invading the spinal cord. It is also interesting to note that many of these structures were found on the border with the glial scar, suggesting important interactions at this interface.

The presence of B cells in different stages of maturation and their enrichment of IL-23 mediated signaling (Fig. 6D) lends further support for the scenario described above, as IL-23 drives germinal center formation [59]. In other tissues, ectopic lymphoid follicles resolve after antigen clearance [80]. It would be interesting to examine spinal cords 90 dpi and beyond to determine when, or if, these structures resolve. Persistence of B cells at 60 dpi suggests their ongoing stimulation and antigen presentation. Prior attempts to identify the antigens have been unsuccessful, but showed they likely don’t include myelin basic protein [22]. Recently, GlialCAM was linked to multiple sclerosis pathology and the induction of a robust B cell response that aggravates EAE [81]. It would be valuable to identify antigens stimulating B cells after SCI and determine whether GlialCAM is also involved in SCI.

Intriguingly, other studies have shown lymphopoiesis is impaired in the bone marrow following SCI and B cells fail to mobilize [82], suggesting B cells observed in the injured spinal cord might have a different origin. Indeed, recent studies suggest the meninges, cerebrospinal fluid, and vertebral bone may be a source of B cells entering the injured CNS [83–86]. Our study showed early B cells in the spinal cord parenchyma are computationally similar to those found in the spinal cord meninges [42], underscoring this as a possible source. Identification of the mechanisms whereby B cells enter the spinal cord, and processes associated with B cell development and activation within the injured spinal cord should help define their role in cell toxicity and allow more nuanced targeting of B cells following injury.

Our analysis uncovered several potential interactions between immune cells, specifically, between microglia, neutrophils, and B cells that may contribute to the recruitment, retention, and accumulation of B cells at the injury site. From the combined functional and CellPhoneDB changes identified, we hypothesize altered microglia–neutrophil interactions signal the early invasion of neutrophils [87] that in turn initiates subacute entry of B cells at the site of SCI [88], which is augmented chronically by signals generated by reactive astrocytes and microglia. Identifying the signals initiating these changes is needed to refine and test these hypotheses.

Notably, we uncovered several potential microglial–B cell interactions that will be worthwhile to investigate further. A recent paper by Brennan et al. [7] also identified the Ccl3/Ccl4–Ccr5 pair through receptor–ligand analysis that we found to be potentially involved in microglial–B cell recruitment, although in the context of interactions between microglia (Ccl3) and macrophages (Ccr5). B cells also express Ccr5, which means they could respond to this chemokine signal to be recruited into the tissue. The Brennan et al. study included similar timepoints (uninjured, 7- and 28 dpi) to ours, so we are encouraged by consistencies in our findings. As microglia have been identified at the scar border after SCI, where we saw an accumulation of B cells [75], this strengthens the concept that these theoretical interactions have physiological relevance. While histological analysis was performed on a different strain of mice than the scRNA-seq, the increase in B cell presence over time was common between strains. We cannot exclude the possibility, however, that localization of the B cells may differ across strains. It would be interesting to perform spatial transcriptomics to identify, where the cell populations we identified are located with respect to the injury site over time and to provide further support for the cell interactions identified computationally in this study.

## Conclusions

In this study, we focused on the gene expression changes of microglia and B cells to subacute and chronic SCI using scRNA-seq. The most marked finding was the large accumulation of B cells located at the injury zone that consisted of multiple stages in the B cell lineage, indicating establishment of ectopic lymphoid follicles. By analyzing ligand–receptor pairs, we identified novel potential interactions between microglia and B cells at separate times after injury, which may help recruit, retain, and drive the B cell accumulation. Functional analyses of the cytokines involved, and their cell sources post-injury, have the potential to develop targeted interventions that modulate the immune responses and reduce associated damage to create an improved environment for tissue repair.

## Supplementary Information

**Additional file 1. **CD45^+^ cells isolated for scRNA-seq. Contour plots, which show areas of density in concentric shapes, of all CD45^+^ cells identified by flow cytometry. Each contour plot is from pooled spinal cords isolated at time indicated. Graphs are plotted as forward scatter (FSC) versus side scatter (SSC). FSC reflects the size of the cell, while side scatter reflects the cells’ granularity. The black box on each plot indicates the cells which were selected for sorting for downstream processing for scRNA-seq. Notice that the cells are smaller in size and less granular, which would preclude isolation of some immune cells including macrophages and neutrophils.

**Additional file 2. **Gating and flow sorting parameters for isolating single CD45^+^ cells, using 3 dpi as an example. **A** Unstained spinal cords were used to set the sort gates and to establish single cell isolation parameters. P1 identifies the region of cells of interest. P2 and P3 are forward and side scatter gates to identify single cells and exclude doublets. P5 and P6 are the CD45^+^ cells which were selected for FACS (both hi and lo populations). **B** The same schematic with the 3 dpi sample to show isolation of CD45^+^ cells following SCI.

**Additional file 3. **Analysis of B cells isolated from SCI combined with those isolated from the meninges. **A** UMAP of all immune cells isolated from the spinal cord in our study (uninjured and injured) informatically combined and integrated with the data from Cohen et al. **B** Mapping of subpopulations of B cells (pre B and mature B identified in meninges, lymph nodes (lymphatic), and from SCI.

**Additional file 4. **Confirmation of cell identity and markers of each cluster. **A** For each cell type identified (microglia, macrophages/monocytes (peripheral myeloid), T cells, NK-/T Cells, and B cells), canonical markers were examined via violin plots to confirm their classifications. **B** Cells isolated from the spinal cord were computationally compared to immune cells in the ImmGen database, confirming that most cells isolated were microglia or B cells.

**Additional file 5.** Geneset enrichments for brain and spinal cord microglia. Tables of GO Biological Process enrichments supporting Fig. 4.

**Additional file 6.** List of DE genes for subacute and chronic microglia following SCI supporting Fig. 4.

**Additional file 7. **B cell cluster analysis in tissue sections. **A** Quantification of proportion of mice analyzed with B cell clusters present anywhere within the lesion site. **B** Quantification of the maximum length of B cell clusters through the cord which were present within the tissue. **C** Representative image of an immunohistochemical staining of spinal cord sections for TMEM119 (microglia) and CD45RA (B cells) from an animal 28 dpi.

**Additional file 8.** Heatmap of marker genes for B cell clusters identified in Fig. 5 after isolating and reclustering of the B cells. Clusters are labeled **A**–**D** as identified in the main text.

**Additional file 9.** List of GO Biological Process enrichments from the total dataset at day 0, 7 dpi, and 60 dpi. Full list of enrichments supporting Fig. 6A, B.

**Additional file 10.** GO terms enriched in clusters for T cells, monocytes/macrophages, NK-/T cells and granulocytes. Wilcoxon rank sum test was used to identify markers for each cell type. Hierarchical groupings were performed as in Fig. 6, and a dot plot was generated. The size of the dot indicates the percentage of gene overlap, and the *x*-axis is FDR. Red dashed line indicates FDR = 0.1. See Additional file 15 for additional enrichments and genes associated with the enrichments found in this figure.

**Additional file 11.** List of all pathways identified using Enrichr for microglia and B cells at individual timepoints as identified in Fig. 6C, D.

**Additional file 12.** CellPhoneDB interactions between microglia, B cells, and T cells. CellPhoneDB was used to determine receptor ligand pairs between microglia, B cells and T cells at the uninjured, subacute, and chronic phases of injury. Results are plotted for interactions in which *p *< 0.01. *P* value is represented on the scale below the tables.

**Additional file 13.** B cells are localized both within the lesion and outside of it. Animals that received a moderate contusion injury were sacrificed at 28 and 42 dpi [44]. **A**, **A’**, **B**, **B’** Sections were stained for B cells in green (B220), astrocytes in red (GFAP), and blood vessels/extracellular matrix in turquoise (Laminin A). **A**, **B** B cells which were seen as clusters could be localized to areas inside the lesion (as identified by the GFAP scar border) at each timepoint analyzed. **A’**, **B’** Individual B cells, rather than clusters, were localized outside of the lesion at each timepoint analyzed (arrowheads). Scale bar = 50 µm.

**Additional file 14.** Lists of receptor-ligand pairs identified in the Venn diagrams of Fig. 7D.

**Additional file 15.** GO Biological pathway enrichments identified in Additional file 10.

## Data Availability

Raw and processed data are available for download from GEO (accession: GSE213471). Processed RData files, suitable for exploration within the R programming environment, along with sample interaction code, as well as code for the above analyses, can be obtained from GitHub via our institution website [89]. The data sets supporting the conclusions of this article are included within the article and its additional files.

## Abbreviations

AD: Alzheimer’s disease
ADRB2: Adrenergic receptor β2
ALS: Amyotrophic lateral sclerosis
APC: Antigen presenting cell
BCR: B cell receptor
CCL: C–C motif chemokine ligand
CCR: C–C motif chemokine receptor
CNS: Central nervous system
CSF1R: Colony stimulating factor receptor 1
DAM: Disease associated microglia
DE: Differentially expressed
DMEM: Dulbecco’s modified eagles medium
Dpi: Days post-injury
EAE: Experimental autoimmune encephalitis
FACS: Fluorescence activated cell sorting
FBS: Fetal bovine serum
FCS: Forward scatter
FDR: False discovery rate
GEO: Gene expression omnibus
GO: Gene ontology
HBSS: Hank’s balanced salt solution
I: Immunoglobulin
IL: Interleukin
IRM: Injury responsive microglia
LTB(R): Lymphotoxin β receptor
NK-/T: Natural killer/T cell
Macs: Macrophages
Monos: Monocytes
MHC-I/MHC-II: Major histocompatibility complex
PBS: Phosphate buffered saline
SCI: Spinal cord injury
scRNA-seq: Single cell RNA sequencing
SSC: Side scatter
SCT: Single Cell Transform
UMAP: Uniform manifold approximation and projection
