# Supplementary figures and images for: Single cell profiling of CD45+ spinal cord cells reveals microglial and B cell heterogeneity and crosstalk following spinal cord injury
Source: J Neuroinflammation. 2022 Nov 4;19:266. doi: 10.1186/s12974-022-02627-3 (PMC9635187; doi:10.1186/s12974-022-02627-3)

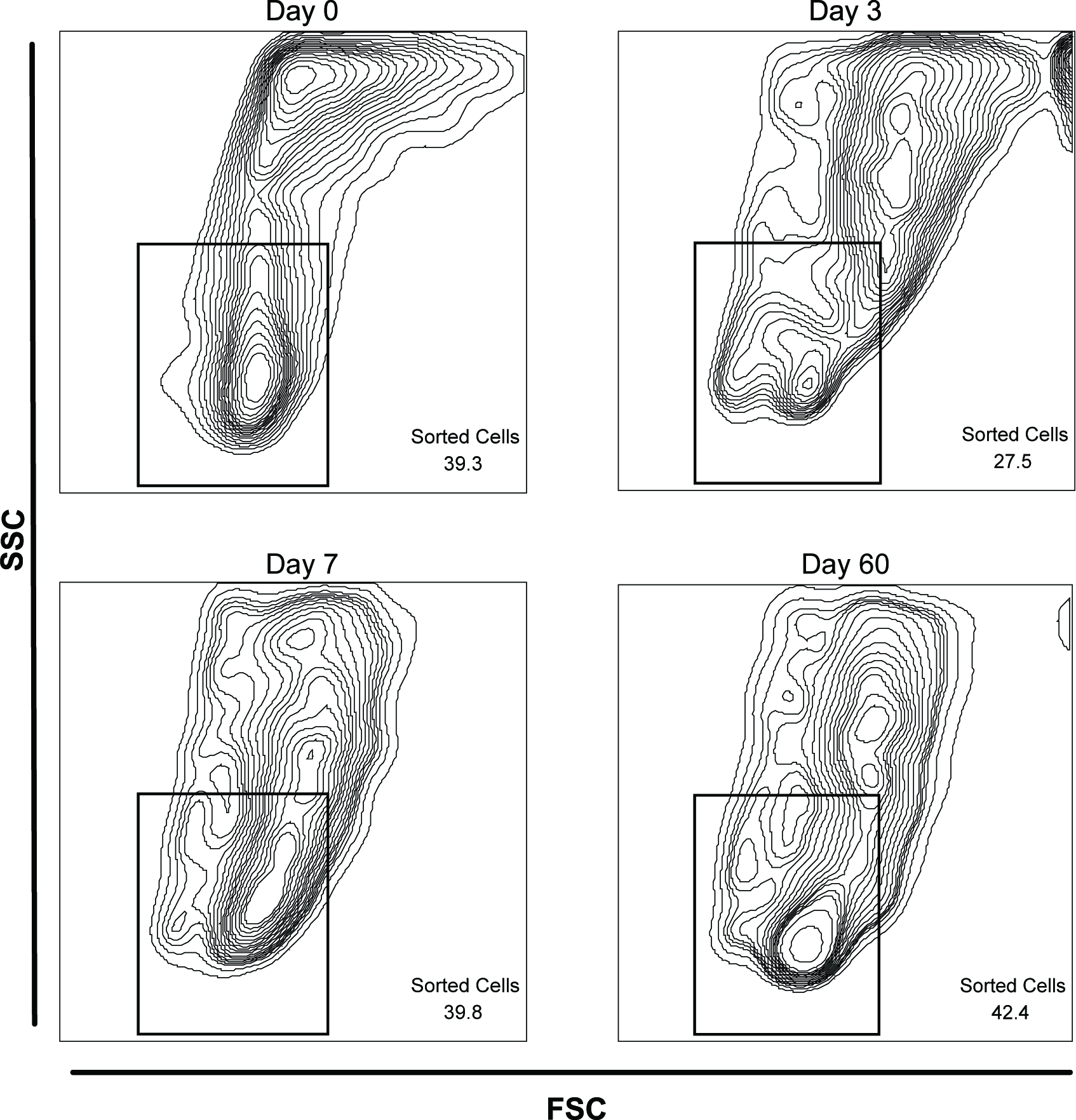

Supplement: Supplementary file 1 — Additional file 1. CD45+ cells isolated for scRNA-seq. Contour plots, which show areas of density in concentric shapes, of all CD45+ cells identified by flow cytometry. Each contour plot is from pooled spinal cords isolated at time indicated. Graphs are plotted as forward scatter (FSC) versus side scatter (SSC). FSC reflects the size of the cell, while side scatter reflects the cells’ granularity. The black box on each plot indicates the cells which were selected for sorting for downstream processing for scRNA-seq. Notice that the cells are smaller in size and less granular, which would preclude isolation of some immune cells including macrophages and neutrophils. [file 12974_2022_2627_MOESM1_ESM.tif]

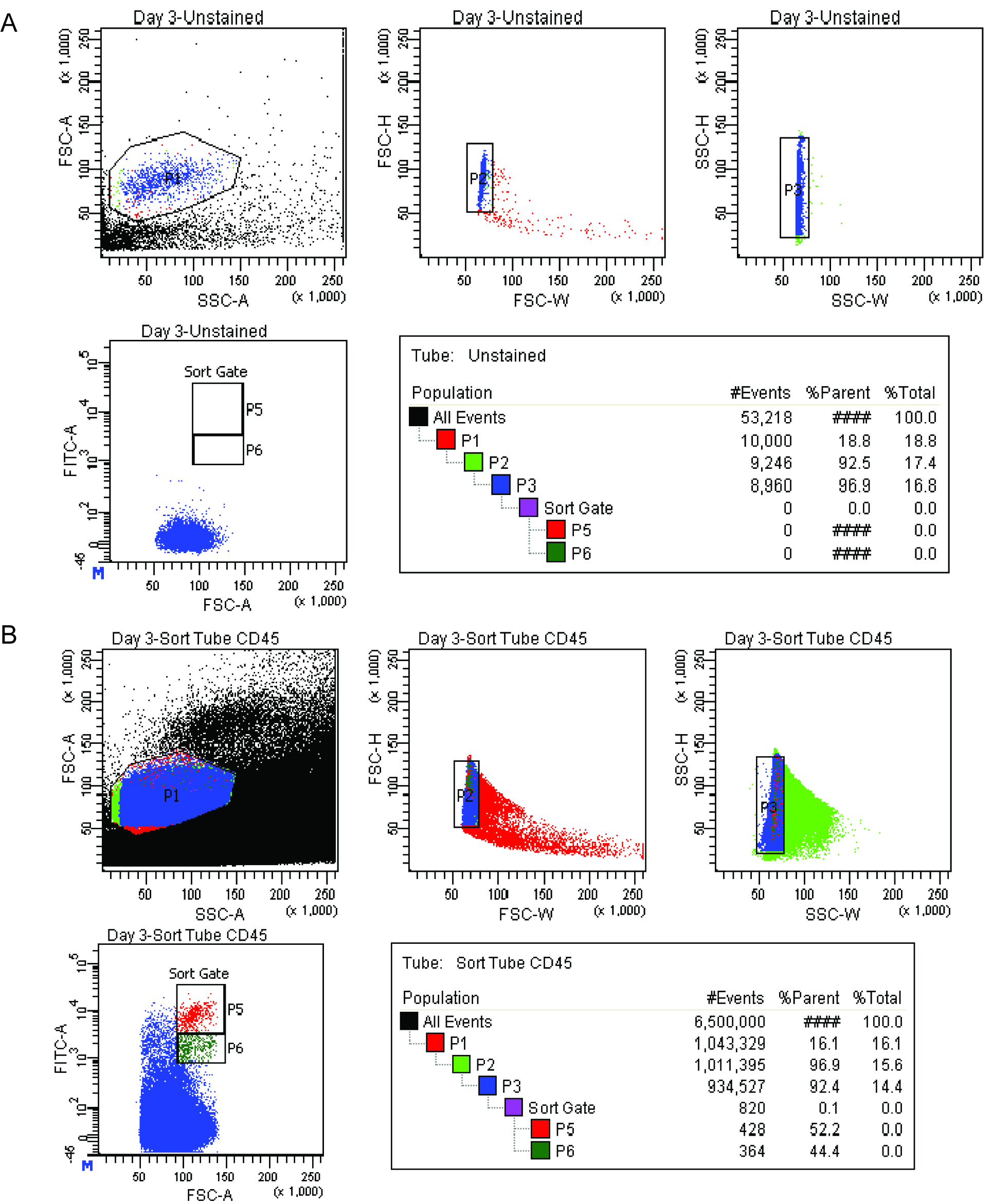

Supplement: Supplementary file 2 — Additional file 2. Gating and flow sorting parameters for isolating single CD45+ cells, using 3 dpi as an example. A Unstained spinal cords were used to set the sort gates and to establish single cell isolation parameters. P1 identifies the region of cells of interest. P2 and P3 are forward and side scatter gates to identify single cells and exclude doublets. P5 and P6 are the CD45+ cells which were selected for FACS (both hi and lo populations). B The same schematic with the 3 dpi sample to show isolation of CD45+ cells following SCI. [file 12974_2022_2627_MOESM2_ESM.tif]

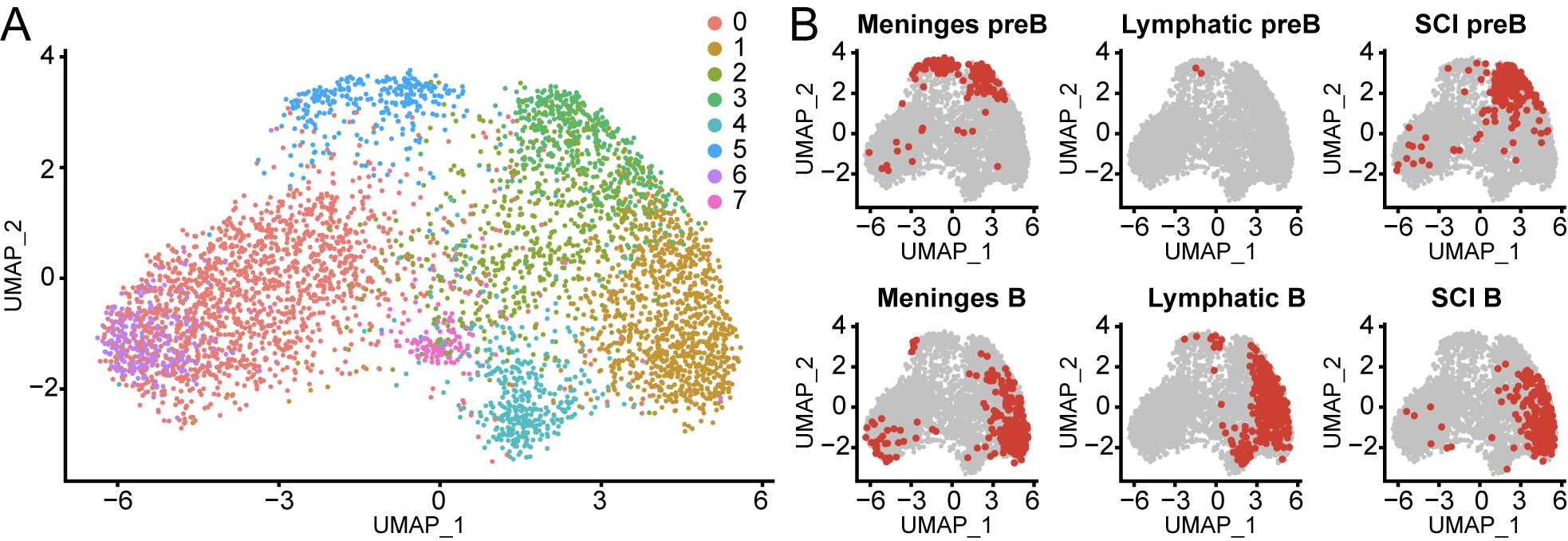

Supplement: Supplementary file 3 — Additional file 3. Analysis of B cells isolated from SCI combined with those isolated from the meninges. A UMAP of all immune cells isolated from the spinal cord in our study (uninjured and injured) informatically combined and integrated with the data from Cohen et al. B Mapping of subpopulations of B cells (pre B and mature B identified in meninges, lymph nodes (lymphatic), and from SCI. [file 12974_2022_2627_MOESM3_ESM.tif]

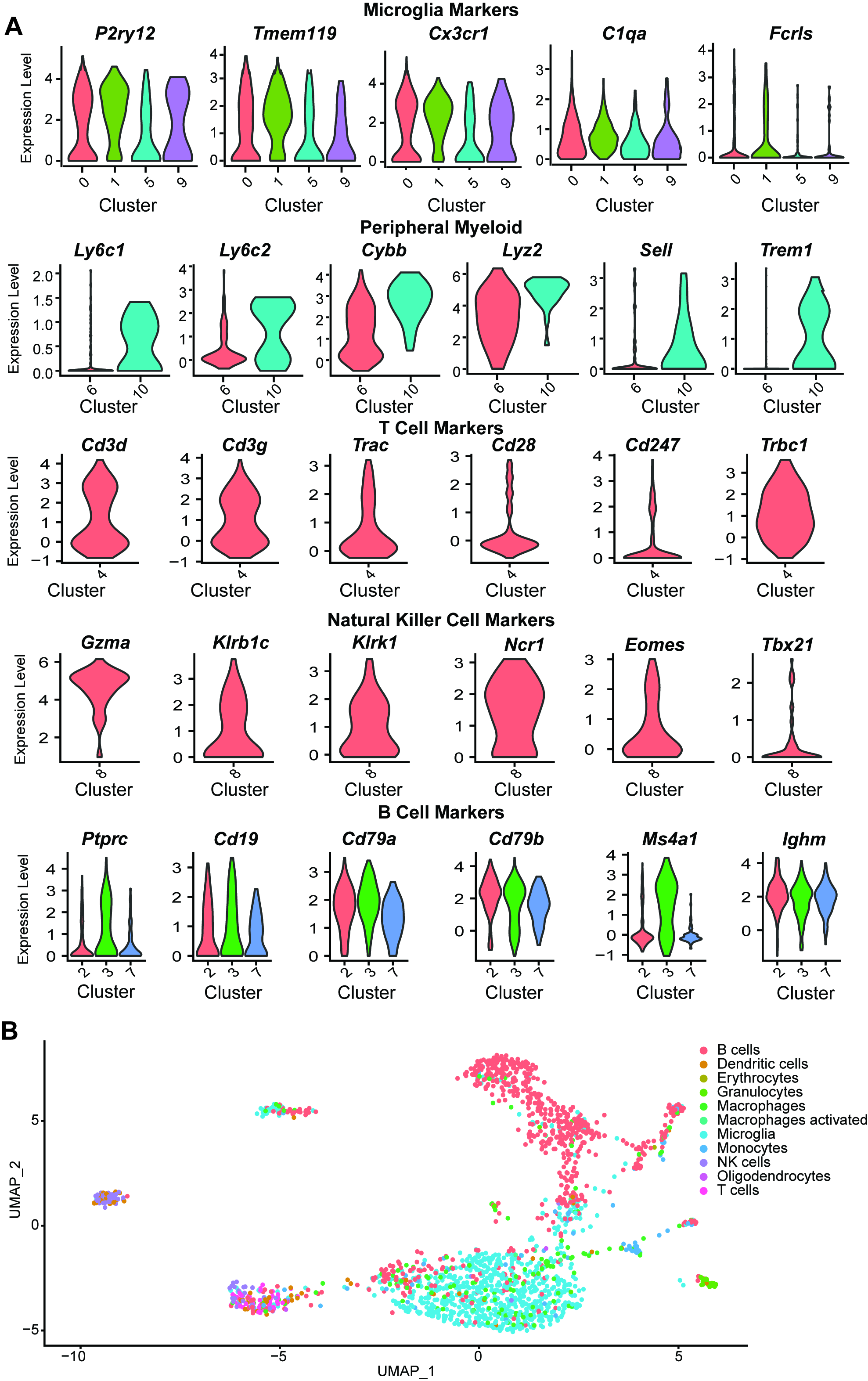

Supplement: Supplementary file 4 — Additional file 4. Confirmation of cell identity and markers of each cluster. A For each cell type identified (microglia, macrophages/monocytes (peripheral myeloid), T cells, NK-/T Cells, and B cells), canonical markers were examined via violin plots to confirm their classifications. B Cells isolated from the spinal cord were computationally compared to immune cells in the ImmGen database, confirming that most cells isolated were microglia or B cells. [file 12974_2022_2627_MOESM4_ESM.tif]

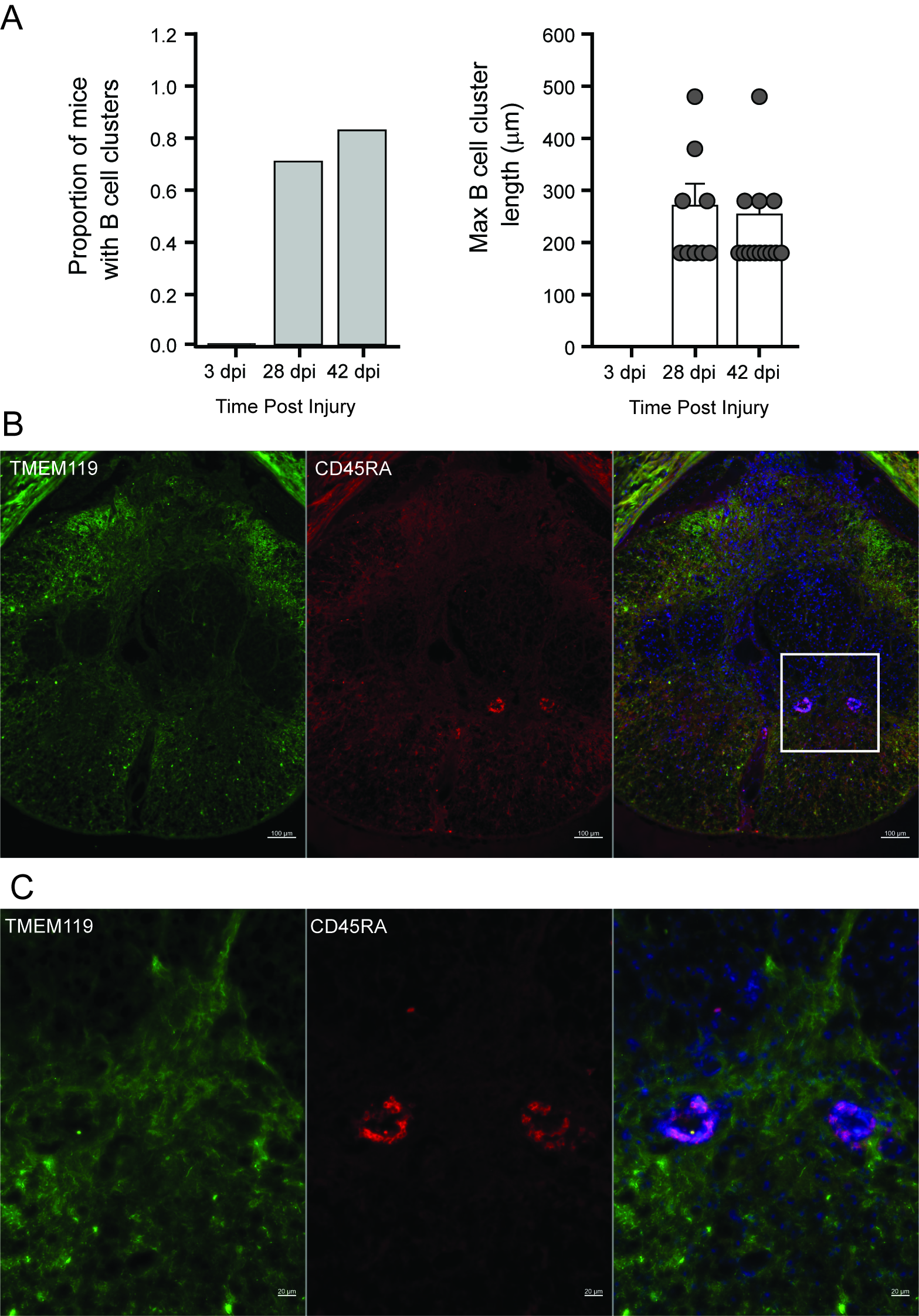

Supplement: Supplementary file 7 — Additional file 7. B cell cluster analysis in tissue sections. A Quantification of proportion of mice analyzed with B cell clusters present anywhere within the lesion site. B Quantification of the maximum length of B cell clusters through the cord which were present within the tissue. C Representative image of an immunohistochemical staining of spinal cord sections for TMEM119 (microglia) and CD45RA (B cells) from an animal 28 dpi. [file 12974_2022_2627_MOESM7_ESM.tif]

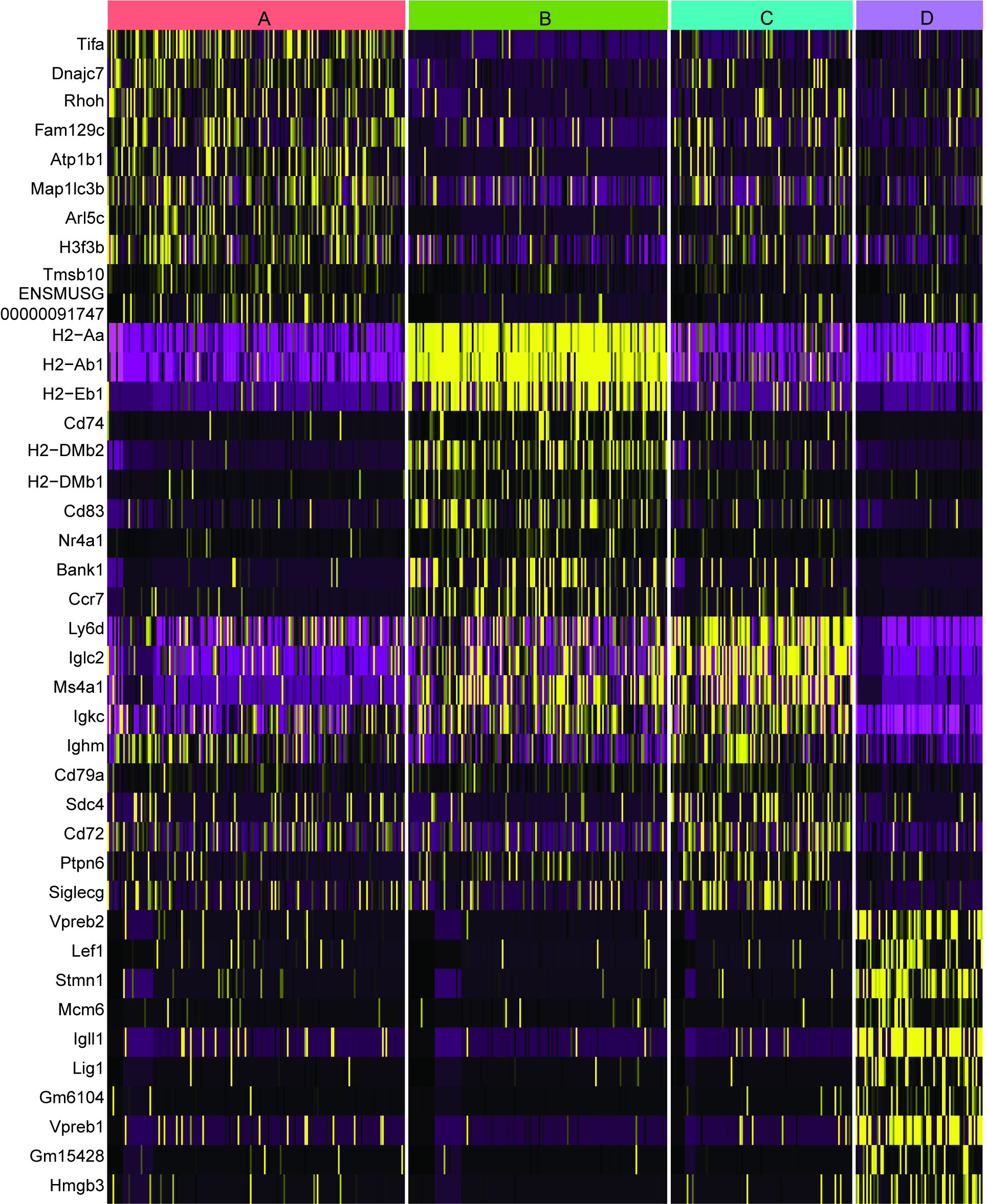

Supplement: Supplementary file 8 — Additional file 8. Heatmap of marker genes for B cell clusters identified in Fig. 5 after isolating and reclustering of the B cells. Clusters are labeled A–D as identified in the main text. [file 12974_2022_2627_MOESM8_ESM.tif]

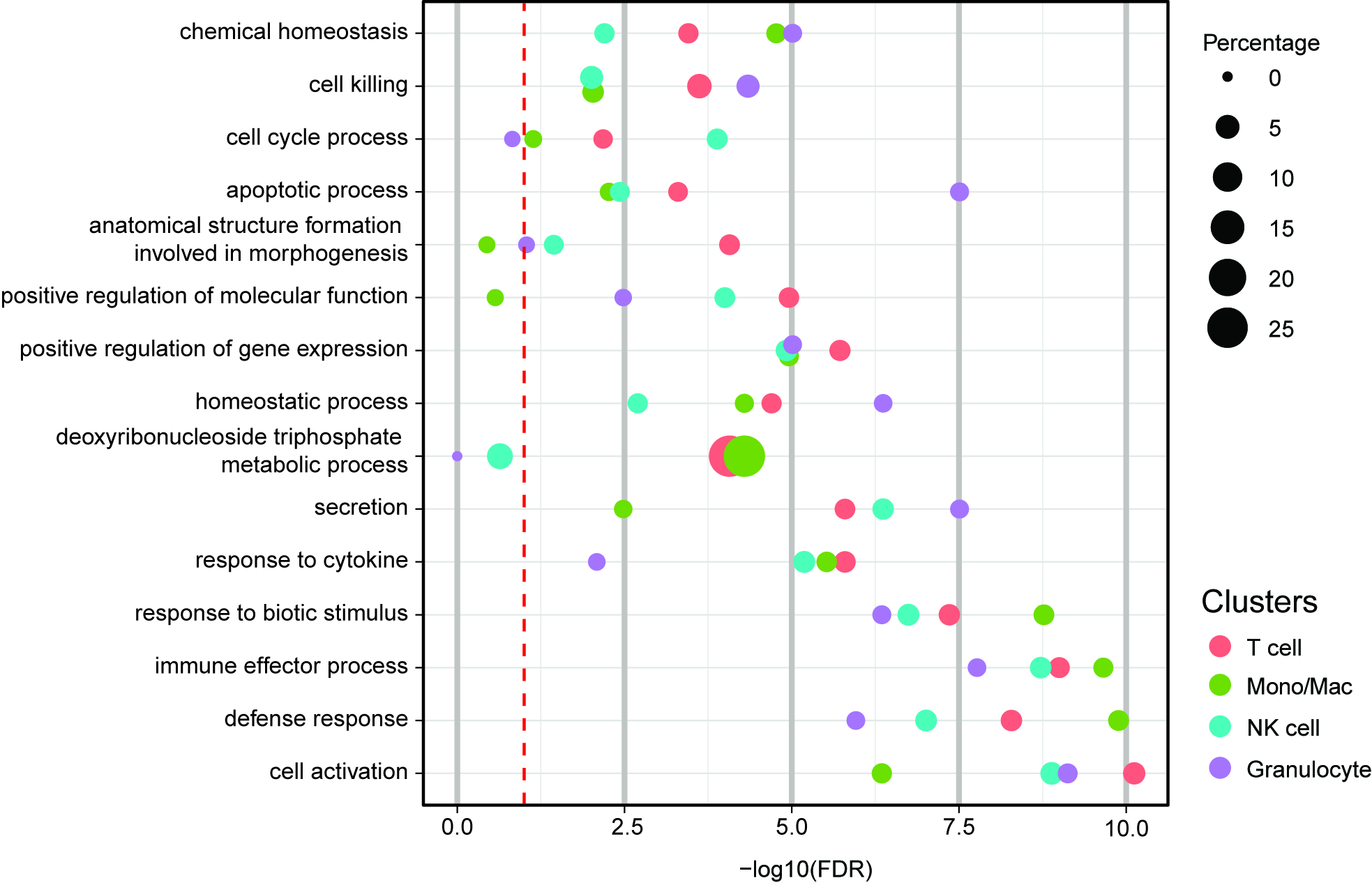

Supplement: Supplementary file 10 — Additional file 10. GO terms enriched in clusters for T cells, monocytes/macrophages, NK-/T cells and granulocytes. Wilcoxon rank sum test was used to identify markers for each cell type. Hierarchical groupings were performed as in Fig. 6, and a dot plot was generated. The size of the dot indicates the percentage of gene overlap, and the x-axis is FDR. Red dashed line indicates FDR = 0.1. See Additional file 15 for additional enrichments and genes associated with the enrichments found in this figure. [file 12974_2022_2627_MOESM10_ESM.tif]

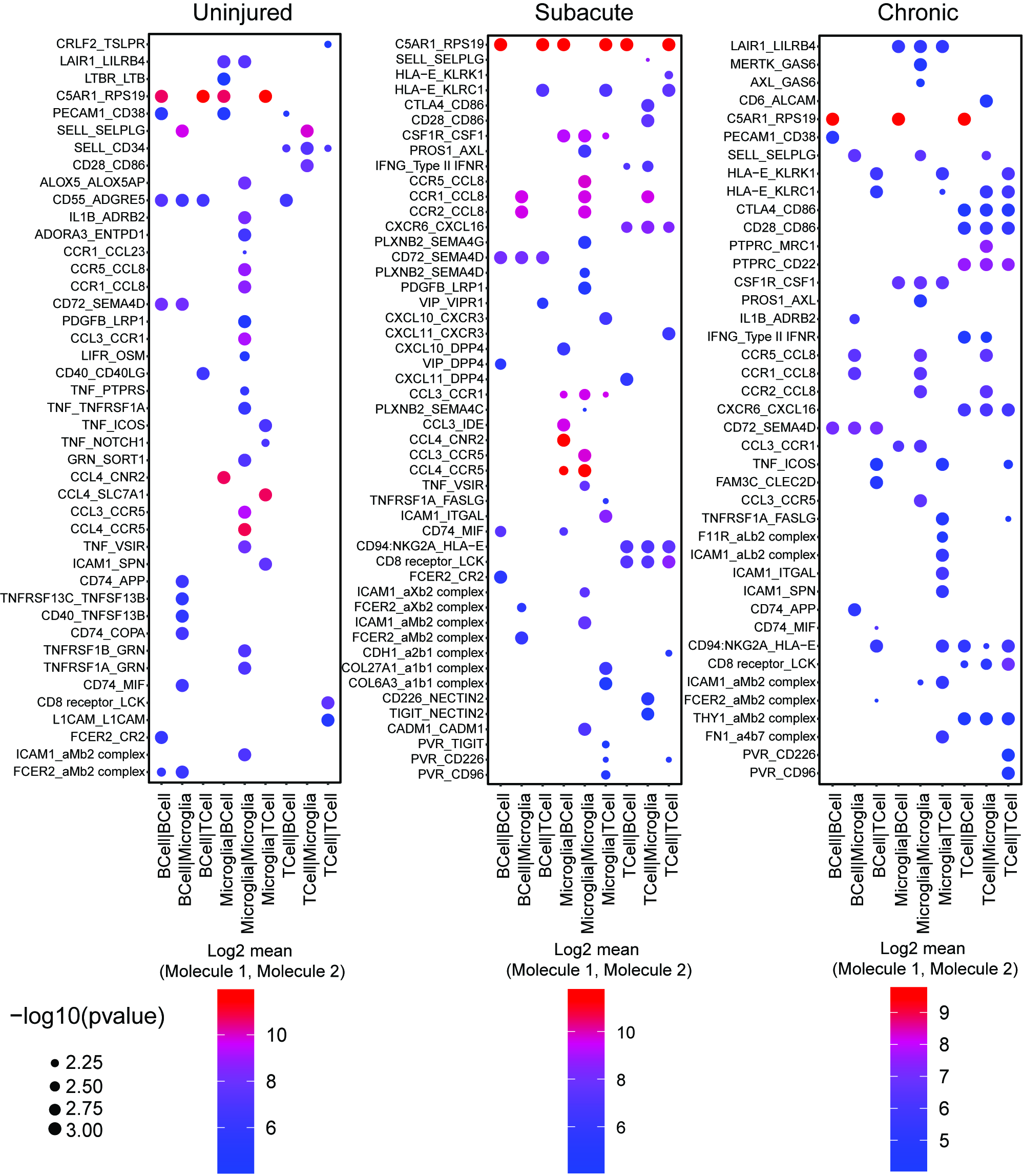

Supplement: Supplementary file 12 — Additional file 12. CellPhoneDB interactions between microglia, B cells, and T cells. CellPhoneDB was used to determine receptor ligand pairs between microglia, B cells and T cells at the uninjured, subacute, and chronic phases of injury. Results are plotted for interactions in which p < 0.01. P value is represented on the scale below the tables. [file 12974_2022_2627_MOESM12_ESM.tif]

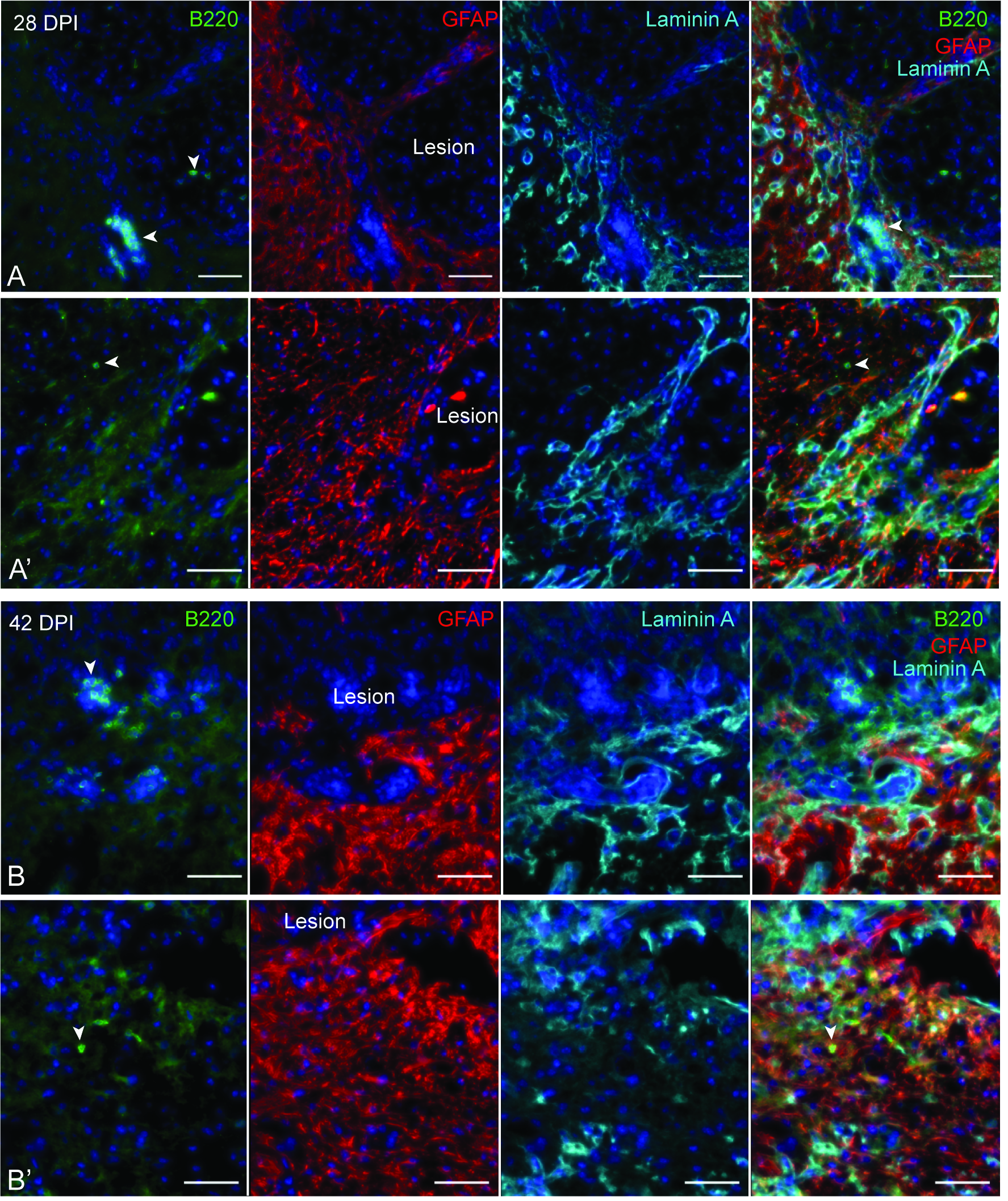

Supplement: Supplementary file 13 — Additional file 13. B cells are localized both within the lesion and outside of it. Animals that received a moderate contusion injury were sacrificed at 28 and 42 dpi [44]. A, A’, B, B’ Sections were stained for B cells in green (B220), astrocytes in red (GFAP), and blood vessels/extracellular matrix in turquoise (Laminin A). A, B B cells which were seen as clusters could be localized to areas inside the lesion (as identified by the GFAP scar border) at each timepoint analyzed. A’, B’ Individual B cells, rather than clusters, were localized outside of the lesion at each timepoint analyzed (arrowheads). Scale bar = 50 µm. [file 12974_2022_2627_MOESM13_ESM.tif]
